# A global collaboration for systematic analysis of broad-ranging antibodies against the SARS-CoV-2 spike protein

**DOI:** 10.1016/j.celrep.2025.115499

**Published:** 2025-04-02

**Authors:** Sharon L. Schendel, Xiaoying Yu, Peter J. Halfmann, Jarjapu Mahita, Brendan Ha, Kathryn M. Hastie, Haoyang Li, Daniel Bedinger, Camille Troup, Kan Li, Natalia Kuzmina, Jordi B. Torrelles, Jennifer E. Munt, Melissa Maddocks, Mary Osei-Twum, Heather M. Callaway, The CoVIC-DB Team, Stephen Reece, Anne Palser, Paul Kellam, S. Moses Dennison, Richard H.C. Huntwork, Gillian Q. Horn, Milite Abraha, Elizabeth Feeney, Luis Martinez-Sobrido, Paula A. Pino, Amberlee Hicks, Chengjin Ye, Jun-Gyu Park, Billie Maingot, Sivakumar Periasamy, Michael Mallory, Trevor Scobey, Marie-Noelle Lepage, Natalie St-Amant, Sarwat Khan, Anaïs Gambiez, Coronavirus Immunotherapeutic Consortium, Ralph S. Baric, Alexander Bukreyev, Luc Gagnon, Timothy Germann, Yoshihiro Kawaoka, Georgia D. Tomaras, Bjoern Peters, Erica Ollmann Saphire

**Affiliations:** 1 Center for Vaccine Innovation, La Jolla Institute for Immunology, La Jolla, CA 92037, USA; 2 Influenza Research Institute, Department of Pathobiological Sciences, School of Veterinary Medicine, University of Wisconsin-Madison, Madison, WI 53711, USA; 3 Carterra, Inc., Salt Lake City, UT 84103, USA; 4 Center for Human Systems Immunology, Departments of Surgery and Integrative Immunobiology, Duke University, Durham, NC 27701, USA; 5 Department of Pathology, University of Texas Medical Branch at Galveston, 301 University Boulevard, Galveston, TX 77555, USA; 6 Galveston National Laboratory, 301 University Boulevard, Galveston, TX 77550, USA; 7 Disease Intervention and Prevention and Population Health Programs, Texas Biomedical Research Institute, San Antonio, TX 78227, USA; 8 Population Health Program, International Center for the Advancement of Research & Education (I-CARE), Texas Biomedical Research Institute, San Antonio, TX 78227, USA; 9 Department of Epidemiology, University of North Carolina at Chapel Hill, Chapel Hill, NC 27516, USA; 10 Nexelis, a Q2 Solutions Company, 525 Boulevard Cartier Ouest, Laval, QC H7V 3S8, Canada; 11 Kymab, a Sanofi Company, Babraham Research Campus, Cambridge CB22 3AT, UK; 12 RQ Biotechnology Ltd., London W12 7RZ, UK; 13 Department of Infectious Diseases, Faculty of Medicine, Imperial College, London SW7 2AZ, UK; 14 Department of Microbiology, University of North Carolina at Chapel Hill, Chapel Hill, NC 27599, USA; 15 Department of Microbiology and Immunology, University of Texas Medical Branch, Galveston, TX 77555, USA; 16 Division of Virology, Institute of Medical Science, University of Tokyo, Tokyo 108-8639, Japan; 17 The Research Center for Global Viral Diseases, National Center for Global Health and Medicine Research Institute, Tokyo 162-8655, Japan; 18 Pandemic Preparedness, Infection and Advanced Research Center (UTOPIA), University of Tokyo, Tokyo 162-8655, Japan; 19 Department of Medicine, University of California, San Diego, La Jolla, CA 92037, USA

**Keywords:** SARS-CoV-2 spike protein, therapeutic antibodies, high-resolution epitope binning, neutralization, coronavirus, COVID-19

## Abstract

The Coronavirus Immunotherapeutic Consortium (CoVIC) conducted side-by-side comparisons of over 400 anti-SARS-CoV-2 spike therapeutic antibody candidates contributed by large and small companies as well as academic groups on multiple continents. Nine reference labs analyzed antibody features, including *in vivo* protection in a mouse model of infection, spike protein affinity, high-resolution epitope binning, ACE-2 binding blockage, structures, and neutralization of pseudovirus and authentic virus infection, to build a publicly accessible dataset in the database CoVIC-DB. High-throughput, high-resolution binning of CoVIC antibodies defines a broad and predictive landscape of antibody epitopes on the SARS-CoV-2 spike protein and identifies features associated with durable potency against multiple SARS-CoV-2 variants of concern and high *in vivo* efficacy. Results of the CoVIC studies provide a guide for selecting effective and durable antibody therapeutics and for immunogen design as well as providing a framework for rapid response to future viral disease outbreaks.

## Highlights

• Features of nearly 400 anti-SARS-CoV-2-spike antibodies were analyzed side by side
• Consortia data are in a publicly accessible database that adheres to FAIR principles
• High-resolution epitope binning defined spike epitope communities with functional importance
• Regression analysis defines a framework for rapid responses to future disease outbreaks

## Graphical abstract

**Figure:**
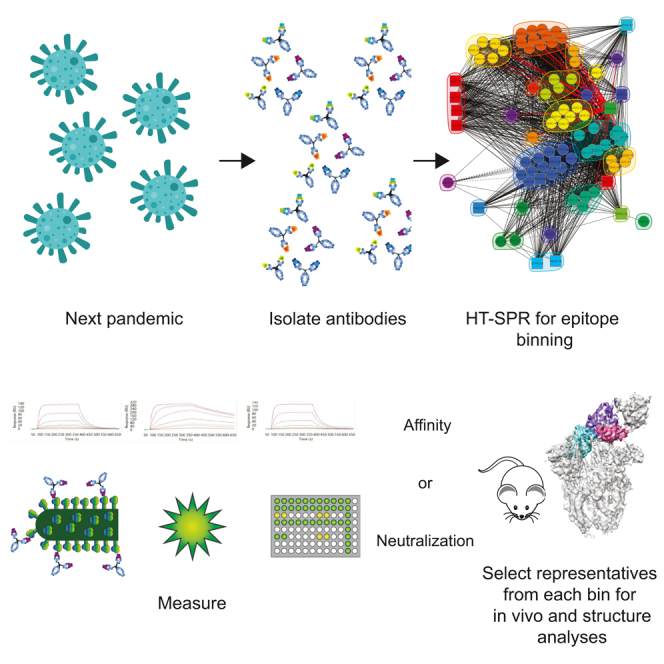

## Teaser

Schendel et al. analyzed ∼400 anti-SARS-CoV-2 spike antibodies using neutralization assays, high-resolution structural analysis and epitope binning, *in vivo* protection studies, and assays for immune escape risk. High-resolution epitope binning defined epitope communities to guide cocktail selection, and regression analysis produced a framework for rapid response to future disease outbreaks.

## Introduction

Nearly 1 billion reported COVID-19 cases and over 7 million deaths have been reported.^1^ Despite effective vaccines,^2^^,^^3^^,^^4^ SARS-CoV-2 infections continue to occur, each associated with variants of concern (VoCs). Discovery efforts sought to identify monoclonal antibodies (mAbs) of therapeutic or prophylactic value. In November 2020, bamlanivimab and REGN-COV were granted emergency use authorization (EUA).^5^^,^^6^^,^^7^ These cocktails and other individual mAbs were escaped by emerging VoCs, suggesting the need for a unified process to determine escape resistance, cocktail selection, and *in vivo* efficacy.

The Coronavirus Immunotherapeutic Consortium (CoVIC) was launched to compare the wide array of mAb treatment candidates proposed by different organizations, side by side, using standardized assays for independent, apples-to-apples analyses. The goals of this analysis were to compare mAbs for inclusion in therapeutic cocktails at present and to determine for the future which antibody features best correlated with *in vivo* protection, which *in vitro* assays predicted protection, and which antibody candidates maintained activity against VoCs and to shape a streamlined process for cocktail selection.

## Results

### CoVIC antibody panel and workflow

The CoVIC collected 407 therapeutic mAb candidates from 61 contributors in industry, academic, and government settings. The majority were contributed from industry groups and included nearly all anti-SARS-CoV-2 mAbs approved for human use as well as mAbs from smaller entities. Initially, the entry criteria for antibodies were broad: nanomolar affinity for the SARS-CoV-2 spike protein and a willingness to explore development for global access. Later, candidate antibodies had to neutralize VoCs or have sufficient rationale (e.g., S2-targeted antibodies) for inclusion.

Contributors sent purified antibodies to the CoVIC headquarters at the La Jolla Institute for Immunology, where each antibody was assigned an ID number (e.g., CoVIC-1) and underwent quality control. The antibodies were then sent under the ID numbers to partner reference labs for analysis (Figure S1). All reference lab data were validated and deposited under their CoVIC ID in the CoVIC database (CoVIC-DB; www.covic.lji.org),^8^ which adheres to FAIR (findability, accessibility, interoperability, and reusability) principles. Contributors retained IP and could use CoVIC data for publications or investigational new drug (IND) applications. Code naming helped ensure blinding, fairness, and public access to results. Around two-thirds of the contributors subsequently unblinded their antibodies.

Here, we focus on 357 CoVIC antibodies that are single immunoglobulin G (IgG) products. The remaining 50 submissions involved non-single IgG products, bispecific formats, or multiantibody cocktails for which assay readouts are not straightforward to compare.

### High-resolution epitope binning

#### Binning using the soluble receptor binding domain of the spike protein

**Figure 1:**
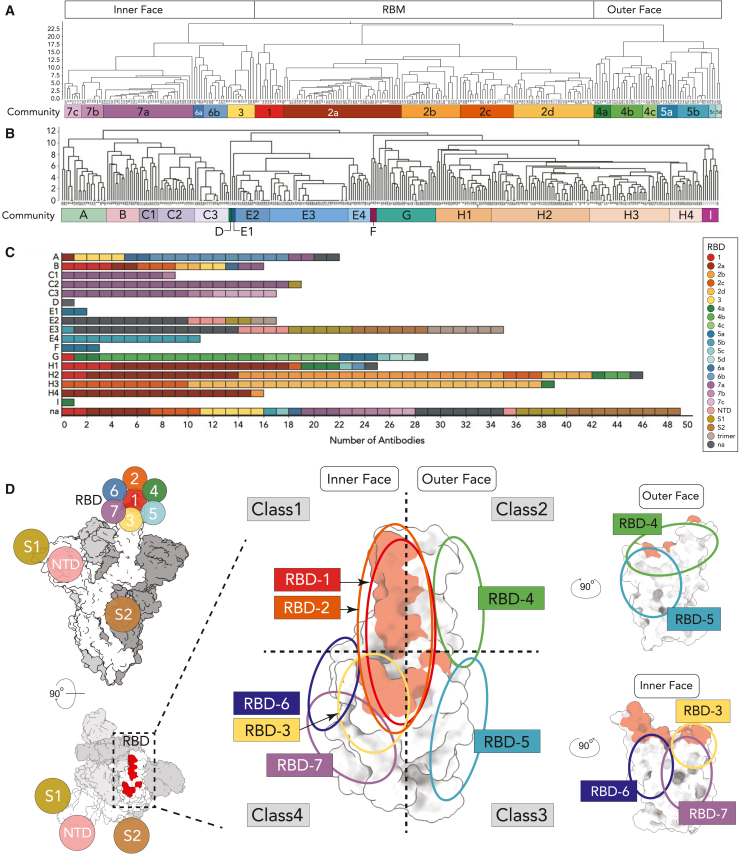
High-throughput surface plasmon resonance (HT-SPR), with the soluble receptor binding domain (RBD) and full-length spike as antigens, defines a broad range of epitope communities (A and B) Dendrograms based on comparisons of competition profile similarity with (A) soluble RBD and (B) full-length spike antigens. Cutoffs were applied to create clusters having highly related profiles. The inner and outer faces, as well as the receptor binding motif (RBM) of RBD are shown. (C) Stacked histogram with full-length epitope communities on the y axis. Individual boxes representing each antibody are shaded according to the RBD epitope community. (D) Location of epitope communities on the RBD. A space-filling diagram of the full-length spike ectodomain with one RBD “up” is shown with relative positions of RBD communities and S1, NTD, and S2 domains indicated. A top view is shown on the lower left with the RBM shaded red. The three monomers of spike are shaded white and light and dark gray. The center shows a space-filling model of the RBD (adapted from PDB: 7A94^11^) with the RBM shaded red. Colored ovals correspond to the general regions targeted by the epitope communities. Vertical and horizontal dashed lines roughly divide the RBD into upper and lower quadrants and an inner and outer face, respectively. Classes defined by Barnes et al. are shown.^10^ The far right shows side views of the RBD.

Early in the pandemic, recognition sites on the spike receptor binding domain (RBD) were roughly divided into four quadrants.^9^ The abundance of antibodies in the CoVIC allowed finer definition of epitope groups through high-resolution, high-throughput surface plasmon resonance (HT-SPR) epitope binning using the LSA platform (Carterra) with either soluble RBD or full-length spike protein as the antigen. Based on pairwise competition, we classified antibodies that bound the soluble RBD into seven main epitope communities: RBD-1–7 (Figure 1; Table S1). The first major division separates communities RBD-3, -6, and -7 (inner face) from RBD-1, -2, -4, and -5. The second major division separates RBD-1 and -2 (receptor binding motif, RBM) from RBD-4 and -5 (outer face). Relative to Barnes et al.’s four-quadrant classification system,^10^ RBD-2 roughly corresponds to class 1; RBD-4 and -5 correspond to classes 2 and 3, respectively; RBD-3, -6, and -7 target sites like those of class 4. Sub-communities for RBD-2 and RBD-4–7 correlate with distinct antibody behaviors within each major group.

RBD-1, -2, -3, -6, and -7 antibodies target epitopes arrayed along the RBD inner face and require the RBD “up” conformation for epitope access (Figures 1A and 1D). Meanwhile, RBD-4 and -5 target epitopes on the outer RBD face that are accessible in both the RBD “up” and “down” conformations. RBD-2 was the largest community (126/357). This relative abundance may reflect early use of soluble RBD as an antibody discovery antigen. Alternatively, the RBD-2 site may be more immunogenic, more exposed, or, if screening efforts focused on antibody function, more likely to neutralize. RBD-7 was the next largest, with many having a multivalent format. RBD-4 and RBD-5, targeting the outer RBD face, have fewer antibodies, yet were divided into three and four sub-communities, respectively, reflecting nuanced binding sites in this region. RBD-3 and -6 were the smallest communities. Interestingly, most RBD-3 members were engineered from SARS-1 antibodies using *in silico* approaches to target cryptic epitopes on SARS-CoV-2 spike. Epitope binning using RBD alone left 79 antibodies unassigned. Of these, 43 were determined or previously known to bind outside the RBD, including the N-terminal domain (NTD), the S2 subunit, other epitopes on S1, or quaternary epitopes on full-length spike (“trimer”).

#### Binning using full-length spike ectodomain

Steric access to epitopes can differ between monomeric RBD and full trimeric spike. Thus, we carried out epitope binning using the HexaPro full-length spike ectodomain (residues 1–1,208),^12^^,^^13^ which identified eight main epitope communities: FL-A–I (Figures 1B and 1C; Table S1).^14^ Reference antibody CR3022^15^ was binned as the only member of FL-D. Communities FL-C, -E, and -H were further divided into sub-communities. We first describe how the RBD communities fit into whole-spike binning.

Most RBD-2 antibodies clustered in FL-H (Figure 1C), while RBD-3 and RBD-6 clustered in FL-A and FL-B. RBD-4 fell into FL-G and FL-H and RBD-5 was distributed across FL-E1, -E4, -F, -G, and -H. Most RBD-7a antibodies were in FL-C, although four, each a VHH (single variable domain on a heavy chain) construct, were FL-A or FL-B. Antibodies in RBD-1 distributed more broadly across four full-length communities (Figures 1C and 1D). Antibodies targeting the NTD binned into FL-E2 or FL-E3, as did antibodies predicted to bind only trimeric spike, and over half the antibodies predicted to bind S1 epitopes that were not binned with the soluble RBD. Six antibodies predicted to bind the S2 region were in FL-E3.

Of the 36 antibodies predicted to bind the RBD but not assigned using monomeric RBD alone, all but one were from convalescent patients. The majority (23/36) binned in FL-E2 and FL-E3 using full-length spike. The remainder were distributed across FL-A, -G, -H1, and -H2 or were not binned. Of the 50 antibodies not binned with the full-length spike ectodomain, over half (57%, 28/50) were binned using the soluble RBD. Together, these results suggest that epitopes exist on the RBD that are accessible only in the soluble form and that the soluble, monomeric RBD has a broader range of epitope exposure than the full-length ectodomain. Moreover, nearly all of the soluble RBD-only antibodies (26/28) require the RBD up conformation.

#### ACE-2 blockage

**Figure 2:**
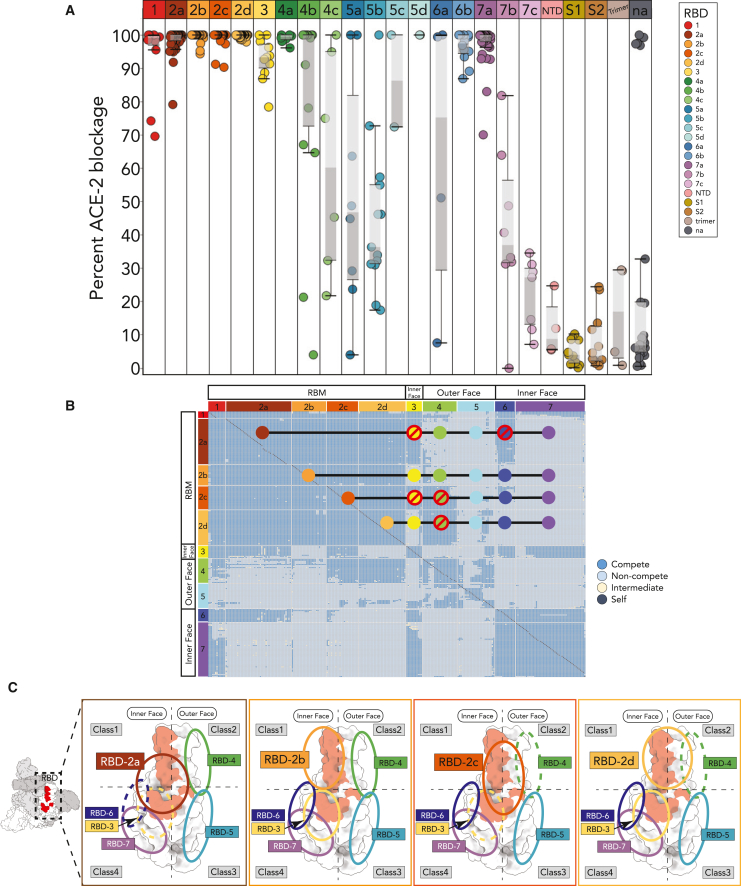
Epitope communities have characteristic degrees of ACE-2 blocking activity and competition (A) Boxplot of the percentage blockage of ACE-2 binding to spike by CoVIC antibodies. The mean value is at the intersection of the darker- and lighter-shaded regions, which represent the lower and upper quartile, respectively. Whiskers extend to 1.5 times the interquartile range. Circles correspond to individual CoVIC antibodies. (B) Competition matrix of CoVIC antibodies, with dark and light blue boxes indicating competition and no competition, respectively, between the antibody pair. Columns and rows represent antibodies as analytes and ligands, respectively. Orange circles represent RBD-2 sub-communities; circles with red lines indicate unfavorable pairing based on competition. (C) Location of epitope communities on the RBD explain, in part, predicted competition between RBD-2 sub-communities and other main communities. The images show top views of the four RBD-2 sub-communities, with dashed ovals indicating epitope communities predicted to compete for binding.

Biolayer interferometry (BLI) was used to measure antibody blockage of interactions between soluble ACE-2 and spike ectodomains (Figures 2A and S2). All but four of RBD-1 and -2 antibodies blocked ACE-2 binding by ≥90%. This result is consistent with the reported antibody germlines (Table S2) in that a high percentage of antibodies with the VH3-53 germline, which is predicted to contribute to high ACE-2 blocking activity,^16^^,^^17^ were categorized as RBD-2 (24/27; 88%). RBD-3 antibodies also had a high degree of blockage, as did RBD-7a members, but RBD-7b and -7c did not. The multivalency of most RBD-7a antibodies may introduce steric hindrance that affects ACE-2 binding higher on the RBD. RBD-7b and -7c members had an IgG1 format, and their lack of blockage would be consistent with their predicted binding site lower on the inner RBD face. Half of RBD-6a antibodies blocked ACE-2 binding, whereas all RBD-6b antibodies had at least 85% blockage, suggesting that RBD-6b epitopes lie higher on the inner RBD face and could impede ACE-2 access. Meanwhile, on the outer RBD face, RBD-4 had varying ACE-2 blocking activity: all RBD-4a and most RBD-4b antibodies blocked binding, but RBD-4c antibodies largely did not. Most RBD-5a and -5b antibodies did not block ACE-2 binding, but both RBD-5d antibodies had 100% blocking. Interestingly, no trimer group antibodies that likely target a quaternary epitope that spans across monomers had appreciable ACE-2 blockage. Most antibodies not assigned using the soluble RBD alone lacked ACE-2 binding blockage. Among antibodies assigned with FL but not RBD, only FL-A, -G, -H1, and -H2 antibodies strongly blocked ACE-2 binding. Antibodies that were unassigned with RBD or FL spike had minimal ACE-2 blocking activity, as did NTD or S2 antibodies. Together, these results show that the high-resolution epitope binning, both with the soluble RBD and with the full-length ectodomain, defines regions on the spike that block ACE-2 binding.

#### High-resolution epitope binning to guide cocktail selection

Arraying the antibodies in a competition heatmap matrix reveals how therapeutic cocktails might be formed (Figure 2B). The four RBD-2 sub-communities have different competition patterns. The RBD-2a footprint lies near the center of the inner RBD face and competes with both RBD-3 and RBD-6. However, RBD-2a can bind at the same time as RBD-7 antibodies, which are predicted to bind lower on the inner RBD face, or with RBD-4 and -5 antibodies against the outer face. RBD-2b competes with only RBD-1 and some RBD-4 antibodies. RBD-2c antibodies can pair with RBD-5 and -7, and some RBD-6 antibodies, but not with members of RBD-3 and -4. RBD-2d can pair with all except RBD-4 and some members of RBD-5. Thus, rather than existing as a single epitope or class, RBD-2 is a continuum across the upper part of the inner RBD face (Figure 2C). Meanwhile, RBD-4, although directed against the outer RBD face, has enough overlap to compete with most RBD-2c and RBD-2d members. RBD-3, which lies lower on the inner face, competes with nearly all members of RBD-2a and -2c, but not RBD-2b or -2d. RBD-7 antibodies compete with those in RBD-6, but largely not with RBD-4 and -5, indicating sufficient epitope separation to allow simultaneous binding (Figure 2C). This finer-resolution binning provides a more detailed guide to select complementary antibodies for a therapeutic cocktail.

#### Neutralization activity of CoVIC antibodies

**Figure 3:**
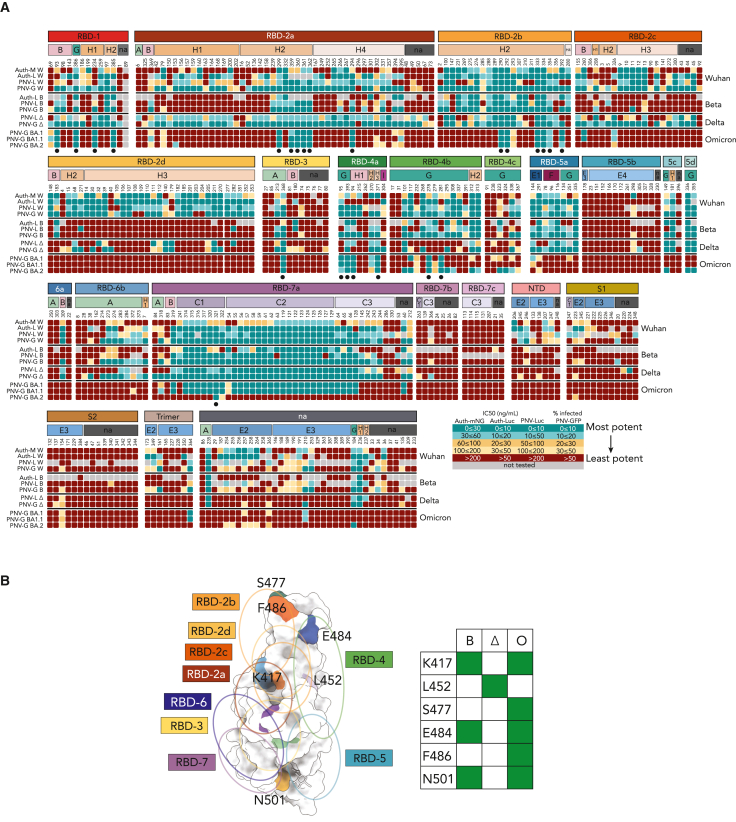
Correlation of results for neutralization assays using pseudovirus and authentic virus and effect of VoCs on CoVIC antibody neutralization activity (A) Neutralization activity for CoVIC antibodies tested with authentic virus carrying mNeonGreen (Auth-M) or luciferase reporter (Auth-L) or rVSV pseudovirus with either luciferase (PNV-L) or GFP (PNV-G) reporters. Neutralization for nearly all antibodies was measured against authentic or pseudotyped Wuhan virus (W). Activity against Beta (B.1.351) was measured for the indicated antibodies using authentic virus with a luciferase reporter and with both pseudovirus platforms. Neutralization toward the Delta variant (B.1.617.2) was tested using both pseudovirus platforms, while neutralization of Omicron (BA.1) and two Omicron sub-variants (BA1.1 and BA.2) was measured using GFP pseudovirus. Shading corresponds to IC_50_ (ng/mL) for Auth-M and -L and for PNV-G. Neutralization of Omicron was measured with 25 μg/mL and 250 ng/mL antibody; the percentage of infected cells using 250 ng/mL is shown. Antibodies are grouped by RBD community and sub-grouped by full-length epitope community with colors corresponding to those in Figure 1. Black dots indicate antibodies that neutralized all VoCs tested. (B) Locations of epitope communities on RBD. Colored ovals correspond to the region targeted by the epitope community. In the table, green boxes highlight the presence of mutations at the indicated residue in Beta (B), Delta (Δ), and Omicron (O) VoCs.

The CoVIC sought to determine whether neutralization assays using authentic virus and pseudovirus displaying SARS-CoV-2 spike returned similar results. We compared results for two vesicular stomatitis virus (VSV)-based pseudovirus assays, with GFP or luciferase reporters, with two assays using authentic viruses engineered to carry a luciferase or mNeonGreen reporter. Overall, there was good correlation (ρ = 0.75–0.88; *p* < 0.05) between authentic and pseudovirus in neutralization success, indicating that, for SARS-CoV-2, pseudovirus represents a suitable surrogate system (Figure 3A; Table S1).

RBD-2, -4b, -5a, and -7a had good neutralization of authentic Wuhan-Hu1 virus engineered with an mNeonGreen reporter (Figure 3A).^18^ RBD-3 can be divided into two groups. Eight are mouse-human chimeric antibodies, which had low neutralization potency. The other RBD-3 (CoVIC-368) had more potent neutralization. Within RBD-4, RBD-4b were generally more potent than -4a or -4c. RBD-4 that were FL-H2 had the most potent neutralization activity and fully blocked ACE-2 binding. RBD-6 antibodies had moderate neutralization potency, with RBD-6b antibodies in FL-A having the highest potency.

Antibodies predicted to bind outside the spike RBD (NTD, S1 outside the RBD, S2, and trimeric spike only) generally had lower neutralization potency, as did antibodies not assigned a community.

Binning with RBD was effective for separating sub-communities having varying degrees of neutralization potency toward Wuhan-Hu1, particularly RBD-5 and RBD-7. Of the four RBD-5 sub-communities, RBD-5a, -5c, and -5d had good potency, while RBD-5b (FL-E4) did not. RBD-5a contains the only two members of FL-E1 and all three FL-F antibodies. Among RBD-7, RBD-7a members, most of which have a multivalent format, overall had the highest potency, particularly for those that binned into FL-C. Within FL-C, FL-C1 and -C2 had higher potency than -C3.

#### Impact of VoCs on neutralization potency of CoVIC antibodies

We also tested neutralization of some antibodies against Beta (B.1.351)^19^ and Delta (B.1.617.2)^20^ VoCs (Auth-L; Figure 3A). For Beta, K417N, E484K, and N501Y affect antibody binding, with K417N and E484K lying on the upper half of the RBD on the inner and outer face, respectively (Figure 3B). N501Y is near the RBD midline. RBD-2a, -2c, and -2d antibody neutralization is largely knocked out for Beta, likely by the K417N mutation. Meanwhile, most RBD-2b members neutralize Beta, likely because the RBD-2b epitope footprint is predicted to lie higher on the inner RBD face than the K417N mutation. RBD-4 and -6 neutralization was also affected to a degree, with RBD-4 particularly vulnerable to the E484K mutation. Meanwhile, RBD-7a neutralization was largely unaffected for Beta, suggesting that the multivalency of most RBD-7a antibodies overcomes the N501Y mutation that lies within the RBD-7a footprint.

The Delta VoC carries an L452R mutation. Neutralization by both RBD-7a and RBD-2a was preserved against Delta. Several RBD-2a (CoVIC-239, -299, and -359–362) antibodies in the FL-H2 group neutralized both Beta and Delta, whereas most RBD-2c antibodies neutralized Delta, but not Beta, in the PNV-G assay. Overall, the RBD-2d sub-community suffered the greatest loss in activity against the Beta and Delta VoCs.

In the RBD-4 community, several RBD-4a antibodies maintained activity against both Beta and Delta, as did some RBD-4b antibodies, which overall had good activity against the Beta sub-variant. Taken together, these results suggest that the RBD-4b antibodies could target the outer RBD face at the midline, where L452R is located, rather than the upper part of the RBD outer face that has the E484K mutation. CoVIC-304, in RBD-4a, is interesting. The sole member of FL-I, CoVIC-304 had the lowest affinity for both spike and RBD among the RBD-4a antibodies and the second-lowest neutralization potency against D614G. However, CoVIC-304 had good potency against Delta, similar to CoVIC-370 and -371 in RBD-4 that neutralized D614G and all VoCs tested. The two RBD-4b antibodies in FL-H2 (CoVIC-312 and -313) had the most potent neutralization activity, and both fully blocked ACE-2 binding. Nearly all RBD-5a, -5c, and -5d members maintained neutralization potency against both Delta and Beta, whereas RBD-5b members had little to no neutralization activity.

Several RBD-6b antibodies neutralized Delta, especially at the higher concentration used in the PNV-G neutralization assay. Although the RBD-6b community was not particularly potent, even against Wuhan-Hu1, the preservation of Beta and Delta neutralization indicates that its epitope is likely conserved. Taken together, most RBD-2a–2c, as well as RBD-5a, RBD-5c, RBD-5d, and RBD-7a members, maintained good activity against the Delta VoC.

The Omicron VoC (BA.1) first emerged in late 2021 and quickly became dominant.^21^ Among the 30 mutations in BA.1 spike, including NTD insertions, 15 are within the RBD. BA.1 shares several mutations with other VoCs (L452, E484, K417, S477, T478, and N501; Figure 3B), while others, like S371L, S373P, S375F, N440K, F486V, and Y505H, occurred less frequently.^22^^,^^23^^,^^24^ We tested the neutralization potency of all CoVIC antibodies at two concentrations (25 μg/mL and 250 ng/mL) against pseudovirus-GFP bearing BA.1, BA1.1, and BA.2. Although 29% (105/357) of the panel neutralized Wuhan-Hu1, Beta, and Delta, only ∼6.7% (24/357) also neutralized Omicron and its sub-variants BA1.1 and BA.2. The Wuhan-Beta-Delta-Omicron-neutralizing (i.e., pan-neutralizing) antibodies were in the RBD-1 (4/13; 31%), -2a (6/49; 12%), -2b (6/27; 22%), -3 (1/11; 9%), -4a (4/8; 50%), -4b (2/15; 13%), and -7a (1/43; 2%) epitope communities. Most pan-neutralizing antibodies were in the FL-H2 and FL-G communities (Figure 3A; Table S1). CoVIC-93, -321, and -371 of FL-B, -C1, and -H3, respectively, also had pan-neutralizing activity. Several antibodies neutralized BA.1 and BA1.1, but not BA.2, and some neutralized only BA.2. Members of RBD-7a, especially FL-C1, -C2, and -C3 members, potently neutralized BA.1 and BA1.1, but nearly all lost neutralization against BA.2, which has T376A and D405N mutations in the RBD-7a footprint. Meanwhile, RBD-4 and RBD-5 antibodies, particularly those in RBD-4b, -4c, and -5d, retained activity against BA.2, which lacks the G446S, G496S, and R346K mutations that lie within their epitope footprints. Interestingly, all but two of these antibodies (CoVIC-312 and -313) are in the FL-G community. Antibodies that retained neutralization activity against the Omicron sub-lineages tested here were also tested by Callaway et al. for neutralization of the BA.4/5 sub-lineage.^25^ In that study, CoVIC-93, -234, -294, -299, -355, and -368 neutralized BA.4/5, but no CoVIC antibodies neutralized subsequent variants like XBB. Again, the high-resolution epitope binning with both the soluble RBD and the full-length spike ectodomain can highlight antibodies that retain neutralization activity against emerging variants.

#### Antibody affinity for spike

SPR binding kinetics analyses showed that the entire CoVIC panel had sub-nanomolar (*K*_*D*median =_ 1.26 × 10^−10^ M) median affinity for the Wuhan-Hu1 (D614G) spike.^14^ All full-length and RBD communities had higher median affinity for the trimeric, full-length spike ectodomain than the monomeric, soluble RBD (both Wuhan) (Figures S3B and S3C). The differences in affinity ranged from ∼6-fold (RBD-6) to over 1,500-fold (FL-I), likely because full-length spike has three available RBD monomers. The RBD-2 and FL-H communities had the best median affinity among the RBD and full-length communities, respectively (*K*_*D*_ = 3.9 × 10^−11^ and 3.6 × 10^−11^ M). Meanwhile, of the RBD communities, the RBD-7b community had the lowest median affinity for full-length Wuhan spike (*K*_*D*_ = 1.9 × 10^−9^ M). The group of antibodies for which no epitope community could be assigned (na) in full-length epitope binning had the lowest median affinity (1.1 × 10^−9^ M).

**Figure 4:**
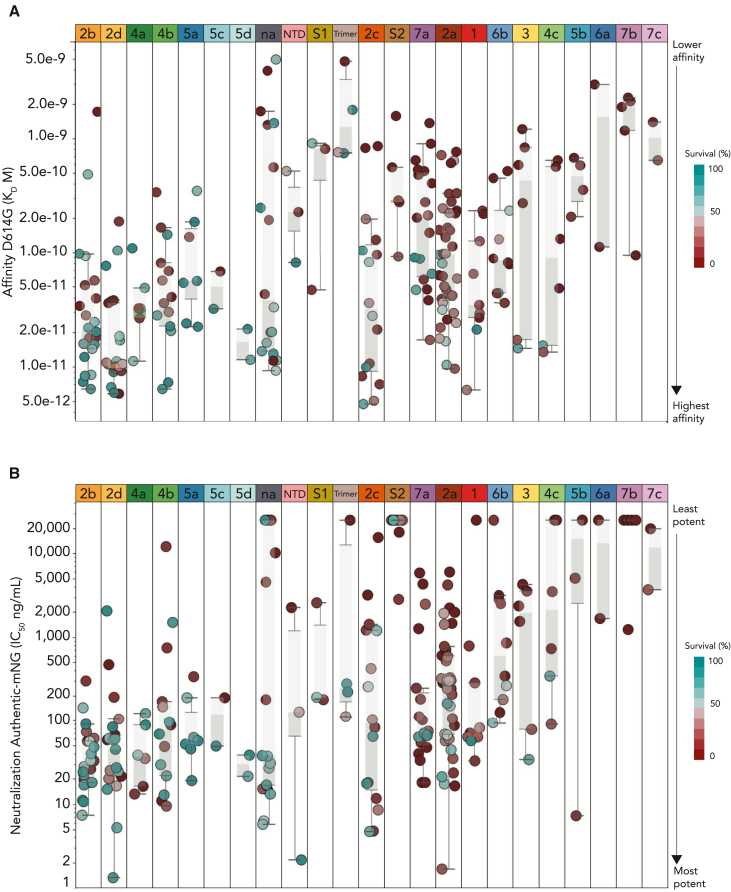
Protective efficacy is related to neutralization and affinity (A) Relationship between RBD community and affinity. Each antibody that was tested in the K18-hACE2 transgenic mouse model of SARS-CoV-2 infection and that had *K*_*D*_ < 1.0 × 10^−9^ M is plotted. (B) Relationship between neutralization of authentic virus with an mNeonGreen reporter (Authentic-M) and protective efficacy. IC_50_ values (ng/mL) are shown on the y axis. Circles in (A) and (B) correspond to individual CoVIC antibodies, with shading corresponding to survival in the K18-hACE2 transgenic mouse model of infection. Dark teal represents the highest protective efficacy. Antibodies were tested using groups of 10 mice with daily monitoring of body weight. Survival is expressed as the percentage of mice surviving at 10 days post-infection. Epitope communities are sorted by the percentage of antibodies within the community that offered at least 60% survival. In the box plots, the mean value is at the intersection of the darker and lighter-shaded regions that represent the lower and upper quartile, respectively. Whiskers extend to 1.5-times the interquartile range.

Around 83% of CoVIC antibodies had affinity for the full-length Beta VoC and 33% bound Omicron (Figures 4A and S3). RBD-1, -4a, -4c, -5b, -5d, and -6b; S1; and S2 had <10-fold reduction in affinity between full-length Wuhan and Omicron, while the median affinity for RBD-2d dropped by ∼3,500-fold. FL-E1, -E3, and -E4 had <∼5-fold reduction in median Omicron affinity. Interestingly, some antibodies, particularly the RBD-7a/FL-C2 group, lost measurable affinity yet still neutralized at least BA.1 and BA.1.1.

Among antibodies that neutralized one or all three of the Omicron sub-variants tested, most lost affinity for Omicron relative to Wuhan (Figure S3). However, CoVIC-364, specific for trimeric spike, had higher affinity for Omicron than other variants (7.51 × 10^−10^, 3.3 × 10^−10^, and 2.4 × 10^−10^ M for Beta, Delta, and Omicron, respectively). CoVIC-333 (RBD-2a and FL-H2) had similar affinity for the three sub-variants (6.4 × 10^−12^, 1.5 × 10^−11^, and 1.9 × 10^−11^ M). RBD-4/FL-G members also tolerated Omicron mutations without substantial loss of either affinity or neutralization activity (Figure S3A; Table S1). The lack of competition between RBD-2a and -2b with most RBD-4a and -4b members suggests that a cocktail comprising antibodies from these communities might retain efficacy against Omicron and its sub-variants (Figure 2B).

#### Relationship of *in vivo* protection with affinity and *in vitro* neutralization

**Figure 5:**
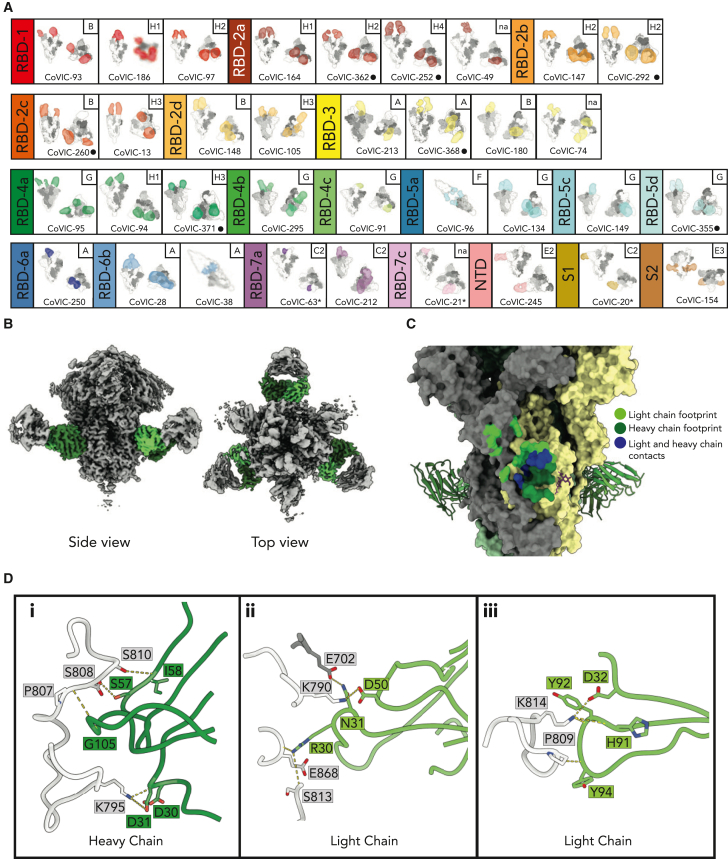
Representative negative-stain electron microscopy (nsEM) structures show the variety of epitope footprints and binding mechanisms (A) nsEM structures of CoVIC panel antibodies in complex with the full-length spike ectodomain were determined. The epitope footprint is shaded by RBD epitope community, and the full-length epitope community is in the upper right of each square. Side (left) and top (right) views of the spike protein are shown. Structures were determined using full-length IgG, except for those with an asterisk by the CoVIC ID, for which Fab or ScFv was used. Black circles indicate antibodies that exhibit bivalent binding. (B) Side and top views of the CoVIC-154-spike complex. The CoVIC-154 variable domain is colored green. (C) Side view of CoVIC-154 Fab bound to spike. Two of the three Fab variable domains are modeled as green ribbon diagrams. The third binding site is illustrated as the antibody footprint on the spike surface. The light chain footprint is illustrated in light green (upper left side), the heavy chain footprint is in dark green, and residues contacted by both chains are in blue. (D) Hydrogen bonding between spike monomers and (i) heavy and (ii and iii) light chains of CoVIC-154. Residues participating in hydrogen bonding are labeled. Spike monomers 1 and 2 are shown in light and dark gray, respectively, and the heavy and light chains are shown in dark and light green, respectively.

The protective efficacy of a sub-set of antibodies (258/407; 63%) was tested in a mouse model of infection using K18-hACE2 transgenic mice expressing human ACE-2 under the control of the epithelial cell cytokeratin (K18) promoter, with weight loss and survival as a metric for morbidity and mortality, respectively (Figure 4; Table S1). CoVIC antibodies were delivered intraperitoneally at 0.5 and/or 1.5 mg/kg doses 24 h before infection with SARS-CoV-2/US WA-1 (GenBank: MN985325). A small sub-set, mostly those predicted to target S2, was tested at 5 mg/kg. Consistent with CoVIC’s original goal of developing potent antibody-based therapeutics for deployment in low- and middle-income countries, the dose was stringent. At these low doses, most antibodies tested offered <70% protection, but 14/258 (∼5%) had 100% protection at 1.5 mg/kg, and one, RBD-5a CoVIC-96, had complete protection at the 0.5 mg/kg dose. This high efficacy at lower concentrations may be associated with an ability to cross-link adjacent spikes (Figure 5A).^26^ RBD-2, -4b, -5a, and -5c each had completely protective antibodies. Most members of RBD-7 were not protective, despite their potent neutralization activity.

Boxplots of affinity for full-length D614G spike ectodomain or neutralization of authentic virus using the mNeonGreen reporter both showed that antibodies with high affinity (Figure 4A) and/or high neutralization potency (Figure 4B) were more likely to offer high protection efficacy. Notable exceptions were antibodies predicted to bind only the trimeric spike, two of which had high protection despite having only nanomolar affinity and moderate neutralization. CoVIC-41 was unique in offering 100% protection (1.5 mg/kg dose) despite having no measurable neutralization. This antibody was isolated from a convalescent COVID-19 patient and had no affinity for the soluble RBD, but had good affinity for full-length D614G, Beta, and Omicron full-length spike ectodomain.

#### Structural analysis of CoVIC antibodies

Structures for 68 CoVIC antibodies were obtained as representatives of each RBD sub-community (except RBD-5b and NTD, S1, and S2) using negative-stain electron microscopy (nsEM; Figures 5A and S4). For expediency, most structures were obtained using intact IgG bound to the full-length spike ectodomain. Use of intact IgG suggested that CoVIC-96 (RBD-5a and FL-F) functions by cross-linking adjacent spike proteins (Figure S4). A bivalent mechanism that likely contributes to maintenance of binding and neutralization of Omicron with its array of mutations was also revealed by using intact IgG.^25^

#### A high-resolution structure for an antibody targeting the fusion loop in the S2 domain

The S2 domain is conserved among human coronaviruses (63%–98% sequence similarity).^27^ Antibodies targeting the S2 domain appear to target three main epitopes: (1) residues ∼1,140–1,160 within the connector domain (CD) proximal to the virus membrane,^28^^,^^29^^,^^30^^,^^31^^,^^32^ (2) the flexible hinge (aa ∼980–1,006) that transitions from a bent hairpin to an extended α helix when the spike protein springs from a pre- to a post-fusion conformation,^27^ and (3) the fusion peptide (∼aa 815–835) near the border between the S1 and the S2 domains.^33^^,^^34^^,^^35^^,^^36^ Structures are available for several S2 antibodies, but most involve a complex between the Fab domain and a linear peptide corresponding to the spike epitope.

CoVIC-154, derived from Wuhan spike immunization of human B cell immune-repertoire Kymice,^37^ is predicted to bind the S2 domain and has several interesting biochemical features. Here, we describe a 2.7 Å cryoelectron microscopy (cryo-EM) structure of the full-length spike ectodomain in complex with the CoVIC-154 Fab fragment (Figures 5B and 5C). Interestingly, the CoVIC-154 Fab bridges two adjacent monomers in the spike S2, with the Fab heavy chain contacting one monomer and the light chain contacting a second monomer (Figures 5C and 5D). Overall, one Fab buries 1,121 Å^2^ total surface area across the two spike monomers and contacts residues that are highly conserved across SARS-CoV-2 variants. The CoVIC-154 Fab heavy chain also contacts the glycan linked at N801.

The CoVIC-154 heavy and light chains both form hydrogen bonds to spike (Figure 5D): from the heavy chain, residues Asp30 and Asp31 to spike residue Lys795, Ser57 to spike residue Asp808, Ile58 to spike residue Ser810, and Gly105 to spike residue Pro807. From the light chain, residue Arg30 bonds to spike Ser813 and Glu868; residues Asp32, His91, and Tyr92 to spike Lys814; residue Asp50 to spike Lys790; and Tyr94 to spike Pro809. Beyond single-protomer interactions, light-chain residue Asn31 also hydrogen bonds to spike Glu702 of a neighboring protomer. The CoVIC-154 Fab epitope overlaps the TMPRSS2 cleavage site (residues 809–821), suggesting that antibody binding may block spike processing and hamper transition to the post-fusion conformation, as suggested by the involvement of residues (e.g., Asp808 and Lys814) at the fusion loop N terminus (Figure 5D, i).

#### Escape-mutation assay detected mutations that later emerged in Omicron

**Figure 6:**
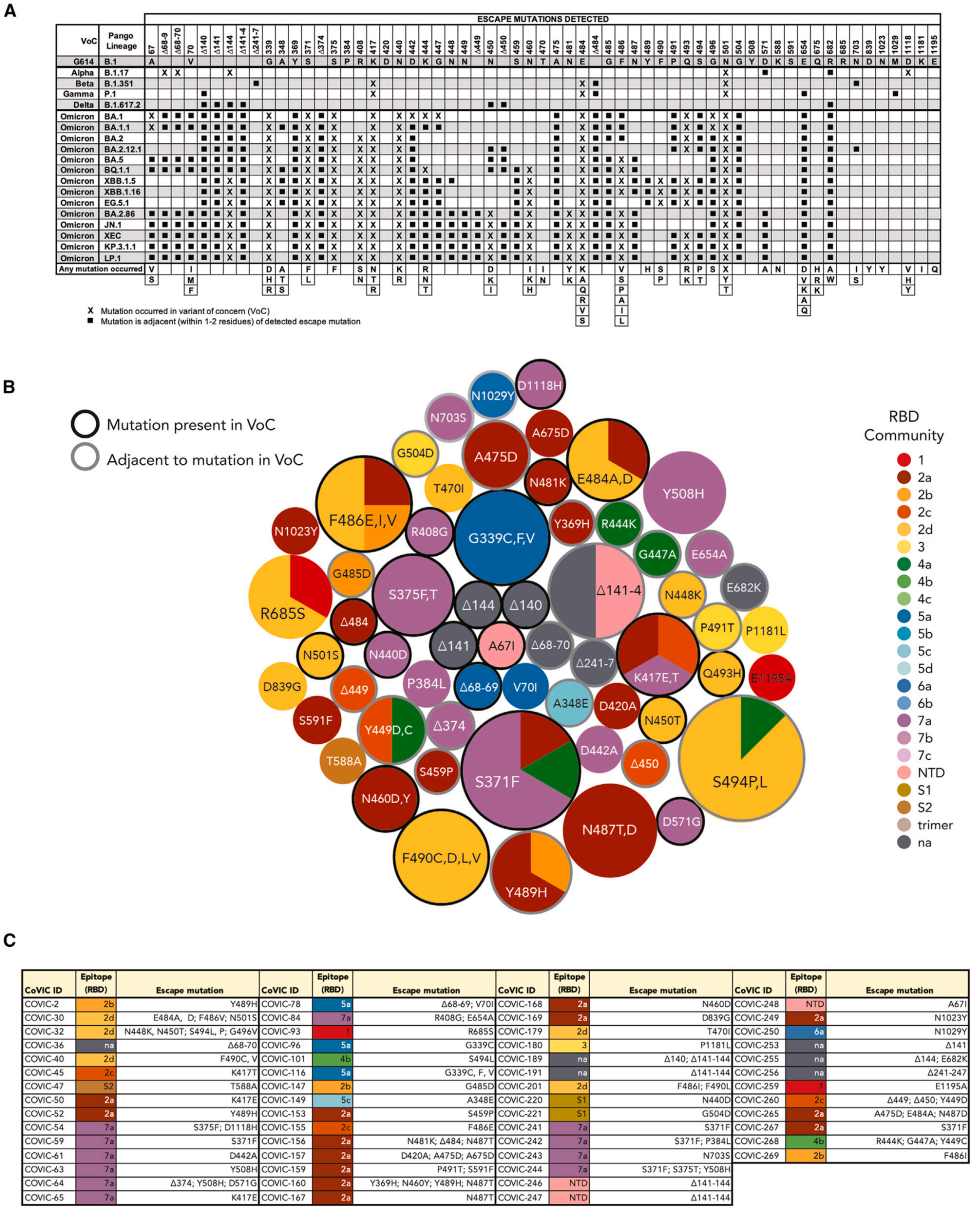
Escape-mutation assay shows regions on spike vulnerable to antigenic escape To identify escape mutations, antibodies were incubated with authentic virus, and the mixtures were added to Vero E6/TMPRSS2 cells. Virus was harvested and the spike gene was sequenced. (A) The top row lists all the amino acid positions at which mutations were detected. Amino acids present in the Wuhan strain (G614, B.1) are shown in the second row, and the rows below list the indicated variants. “X” indicates that the residue was mutated in a variant. Squares indicate that the detected mutation was within one or two residues of an amino acid mutated in a variant. Rows at the bottom show mutations reported in public databases (e.g., GSAID). (B) Bubble plot of detected escape mutations. Each circle shows the residue that was mutated (some residues had more than one amino acid change detected). Circle size corresponds to the number of antibodies affected by the mutation. Circle color indicates RBD epitope community; some mutations affected multiple epitope communities and the circle is divided according to the percentage of each epitope group affected. Residue numbers radiate outward, with the lowest residue number in the center. (C) Detected escape mutations.

At the CoVIC study outset, months before major VoCs emerged, we used an assay to detect areas on spike vulnerable to antibody escape.^38^ This assay detected 76 unique mutations at 52 different sites, including 11 deletion mutations and 65 point mutations (Figures 6 and S5). Most mutation sites (71%) localized to the RBD. The NTD had seven deletions and two point mutations (A67I and V70I). Sub-domains 1 and 2 (SD1 and 2), the central helix (CH), the CD, and the heptad repeat (HR2) also had mutations. The assay detected mutations at RBD residues E484 and N501, which were mutated in both Beta and Gamma. A deletion mutation (Δ68–69) and point mutation (D111^∗^) in the Alpha variant were also detected. Four different mutations localized to sites adjacent (within one or two residues in the linear amino acid sequence) to the L452 mutation in Delta. Of the 34 mutations in Omicron, we detected 12 sites, plus another 12 adjacent to mutation sites (Figures 6A and S5). Notably, the N481K mutation, carried by more recently emerged VoCs, BA.2.86 and JN.1, but no previous major VoC, was identified. Together, the results indicate the potential of the escape assay to detect areas on spike vulnerable to mutations that could impact neutralizing antibody binding.

#### Guidance for selection of antibody candidates for future disease outbreaks

The CoVIC data provide an opportunity to determine, at scale, what antibody features correlate with *in vivo* protection. We carried out a regression analysis considering: (1) RBD and FL epitope community, (2) D614G binding affinity, (3) neutralization of pseudovirus with luciferase (PNV-L) or GFP (PNV-G) reporters, (4) neutralization of authentic virus with luciferase (Auth-L) or mNeonGreen (Auth-M) reporters, and (5) blockage of spike-ACE2 binding. This analysis included data and *in vivo* protection results for 214 CoVIC antibodies.

**Figure 7:**
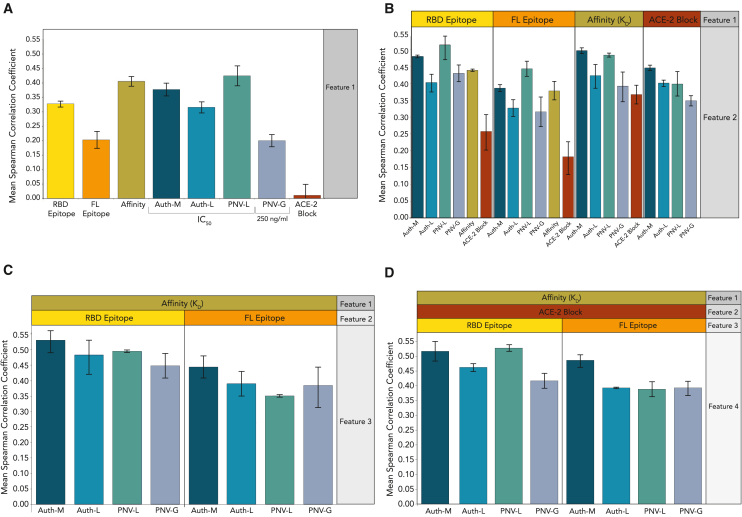
Five-fold cross-validation using different combinations of antibody features to predict *in vivo* protection The analysis was carried out on a sub-set of antibodies for which *in vivo* data and all seven features considered were available. Mean Spearman’s correlation coefficients over three replicates are shown; error bars indicate SEM. (A) Plot of the ability of individual antibody features to predict *in vivo* protection. Then, (B) two, (C) three, or (D) four features were combined to determine whether the predictive performance was enhanced. Auth-M and Auth-L indicate neutralization of authentic virus with mNeonGreen and luciferase reporters, respectively. PNV-L and PNV-G indicate neutralization of pseudovirus with luciferase and GFP reporters, respectively. The PNV-G assay tested a single concentration (250 ng/mL), and the other neutralization assays reported IC_50_ values determined from eight-point curves. RBD and FL are epitope communities determined from binning with soluble RBD and trimeric full-length spike ectodomain. Affinity is the *K*_*D*_ (M) for the D614G full-length ectodomain and ACE-2 block is the percentage blockage in the presence of antibody.

For the regression analysis, we applied the ensemble method CatBoost,^39^^,^^40^ involving gradient boosting on decision tree outputs wherein one iteration is used to improve decision tree results in the next iteration. Using 5-fold cross-validation, we first evaluated how individual features predicted *in vivo* protection by calculating Spearman’s correlation coefficient between the predicted scores of a given antibody using only one feature as an input and its actual *in vivo* protection. The PNV-L had the highest correlation between predicted and actual protection (PNV-L, ρ = 0.42; Figure 7A), followed by D614G affinity (ρ = 0.40, Figure 7A). Epitope communities based on soluble RBD consistently had higher correlation in predictive power than those defined using full-length spike (ρ = 0.33 vs. 0.20, Figure 7A).

We next tested whether combinations of antibody features improved predictive performance. The RBD epitope combined with PNV-L (ρ = 0.514) had the best performance, followed by Auth-M combined with D614G affinity (ρ = 0.5). Neutralization of Auth-M combined with RBD epitope community and PNV-L combined with D614G affinity both had slightly lower correlation (ρ = 0.48) (Figure 7B). We obtained a slightly higher correlation with the three-feature combination Auth-M, RBD epitope community, and D614G affinity (ρ = 0.53; *p* > 0.05) (Figure 7C), whereas combining four features did not appreciably increase the correlation with protection (Figure 7D). Based on this analysis, RBD epitope community paired with either pseudovirus neutralization or D614G affinity had the highest predictive value for *in vivo* protection.

Neutralization with a single concentration point was, unsurprisingly, less predictive than a concentration curve. Also, ACE-2 blockage, either alone or in combination, has very little predictive capacity of *in vivo* protection (Figures 7A, 7B, and 7D), perhaps because nearly two-thirds of CoVIC antibodies potently blocked ACE-2 binding yet only around one-quarter conferred *in vivo* protection (Figure 7D).

Our regression analysis suggests that epitope community paired with pseudovirus neutralization or binding affinity provides sufficient information to predict *in vivo* protection, and additional information did not significantly improve such predictions. In the next pandemic, selecting which features to prioritize in a discovery campaign can, to some degree, be determined by the scope of activities or equipment available. For SARS-CoV-2 and these assays, pseudovirus neutralization and authentic virus neutralization were similarly predictive, and detecting neutralization at a single concentration yielded valuable information with higher throughput. For other viruses, authentic virus neutralization assays may be more predictive of protection, as we showed in a field-wide analysis of antibodies against Ebola virus.^41^

## Discussion

The CoVIC study offered the opportunity to compare proposed therapeutic candidates from 61 different discovery groups and companies and the opportunity to draw conclusions at scale and across discovery platforms. The scope of the CoVIC panel allowed definition of detailed epitope communities that had functional relevance in terms of protective efficacy. Here, we addressed whether neutralization *in vitro* forecast protection *in vivo*. CoVIC antibody neutralization indeed correlates with protection *in vivo*, in that almost all non-neutralizing antibodies were non-protective in the K18-hACE2 transgenic mouse model of infection. However, neutralization alone did not guarantee *in vivo* success. Multiple RBD-2, -3, -4, -5, and -7 antibodies neutralized potently, yet failed to protect. Most RBD-7a members were not natural IgG and, despite exhibiting potent neutralization, failed to protect *in vivo*. A pilot study performed in parallel with the *in vivo* assays indicated that this failure to protect was due in part to poor pharmacokinetics.

In general, RBD-2a, -2b, -4a, -4b, -5a, -5c, -5d, and -7a members were more likely to neutralize and more likely to be potent against VoC. Outside of the RBD, neutralization potency was generally weak. A notable exception is CoVIC-154, for which we obtained a cryo-EM structure. CoVIC-154 was the only S2 antibody that had measurable neutralization.

Interestingly, here, both affinity and neutralization were strong correlates of *in vivo* protection. Antibodies with the highest affinity offered, on average, the highest neutralization potency and the highest survival. However, CoVIC-161, -210, and -364, all FL-E3, conferred high levels of *in vivo* protection despite having relatively low affinity for full-length ectodomain spike. These mAbs bind trimeric spike and seem to “punch above their weight” to provide *in vivo* protection greater than their affinity would suggest.

Other pandemics will come, and the discovery of mAbs against surface glycoproteins present on pathogens with pandemic potential will provide opportunities for treatment and direction for vaccines. Together, the CoVIC results suggest an expedient discovery strategy wherein, after rapid discovery, sorting antibodies into fine epitope communities in a competition analysis can define consistently predictable behavior of the antibodies relative to one another. Within each epitope community, the highest-affinity mAbs could be advanced for structural and functional analyses, including *in vivo* protection. Cocktails of antibodies from the complementary communities would allow provision of therapeutics more likely to withstand inevitable mutations. Moreover, our results indicate that nsEM using intact IgG can reveal geometry of recognition, including bivalent binding patterns, which may help maintain neutralization if VoCs emerge,^25^ as well as spike cross-linking, associated here with improved neutralization and *in vivo* potency.

Multiple studies described antibodies and antibody activities throughout the SARS-CoV-2 pandemic. What did we learn here that could not have been learned by considering prior literature alone? First, the 357 different therapeutic candidates analyzed here came from different companies, laboratories, and discovery centers and would not have been compared side by side in other work. These larger-scale analyses afford both apples-to-apples and relative comparison of numerous antibody features. All data are deposited in the publicly available CoVIC-DB (www.covic.lji.org), which adheres to FAIR principles and allows data download for analysis.

The CoVIC scale provided sufficient independent examples of antibody behavior to link particular behaviors to different antibody groups. For example, we had enough RBD-2 antibodies that did and did not neutralize Omicron to discern that antibodies with bivalent spike binding neutralized Omicron, whereas those that bound monovalently to the same site were escaped. Moreover, RBD-5, some of which could cross-link adjacent spikes, had as many sub-communities as the far larger RBD-2 and could be distinguished only through a panel of this size. The size of the CoVIC panel further allowed us to identify three antibodies identified by separate teams, each with lower affinity, but each bound a quaternary epitope associated with greater protective efficacy than affinity alone would suggest.

We also learned that samples not binned with RBD could be binned using the full-length ectodomain spike and vice versa. Across the discovery sites, 35/36 antibodies that could not be binned on RBD alone came from convalescent patients rather than immunization or *in silico* development. We further learned that an *in silico* approach yielded antibodies in an unusual epitope group (RBD-3) rarely accessed by antibodies from immunization or convalescence.

Other work is confirmatory, but confirmatory at great scale and including a broad array of samples not previously subjected to this many assays: across 357 mAbs, the four neutralization assays were essentially equally predictive of *in vivo* efficacy in the K18-hACE2 transgenic mouse model. Neutralization correlates with, but does not guarantee, protection, as many highly potent neutralizers failed to protect. Among the non-neutralizing antibodies, only three, CoVIC-41 (not assigned), -341 (predicted S2), and -391 (RBD-4b and FL-G), conferred *in vivo* protection.

The scale of the study also allowed analysis of which VoCs and point mutations within VoCs knocked out which groups and allowed suggestion of non-competing, mutation-resistant pairings of potent neutralizers with good *in vivo* activity. The results suggest that an RBD-2a or -2b paired with an RBD-4a and -4b should offer a VoC-resistant cocktail. At a large scale, the competition grid and escape maps also offer a publicly available repository of information should a group with a monotherapy wish to find a complementary antibody to make a cocktail.

Finally, the study offers data-driven recommendations on which *in vitro* features can be measured rapidly to offer the greatest predictive value for antibody selection in the next pandemic. Although we cannot know which virus, or even virus family, will cause the next pandemic, this large-scale, side-by-side comparison of assays provides a guide to be better prepared to develop effective medical countermeasures.

### Limitations of the study

This study has several limitations. First, most (91%) antibodies were submitted in the first 18 months of the COVID-19 pandemic, before Delta and Omicron emerged. Antibodies that had potent activity against all three Omicron sub-lineages did not neutralize subsequent variants like XBB.^25^ The panel closed to new submissions in September 2022 and thus would not include broadly neutralizing antibodies that likely emerged in individuals who either were infected with subsequent VoCs or received booster vaccines carrying Omicron sub-lineages. Such antibodies may target antigenic sites that differ from those in this panel or may use a different binding mechanism. Second, the blinded nature of the study allowed antibodies to be analyzed on an equal footing, but the terms of CoVIC submission did not require the contributor to provide information about the antibodies beyond demonstration of nanomolar affinity or, subsequently, VoC neutralization activity. In particular, for some, we could not perform sequence analyses to understand the basis for antibody binding, particularly how mutations in VoC affected antibody function or how antibodies might be engineered to overcome VoC mutations. Third, the *in vivo* protection assay dose was stringent at 1.5 mg/kg and chosen to distinguish the most potent antibodies for deployment in low- and middle-income countries, but this low dose may miss antibodies that have protective activity. The *in vivo* study also considered only female animals. Last, the scope of work for the CoVIC study did not allow testing of antibody combinations to validate predicted competitions or to discover possible synergistic actions of antibodies in combination.

## Resource availability

### Lead contact

Direct requests for further information and resources and reagents to the lead contact, Erica Ollmann Saphire (erica@lji.org).

### Materials availability

Request information concerning particular antibodies through the CoVIC at https://covic.lji.org.

### Data and code availability

This paper does not report original code. All reported data reported are publicly available at the CoVIC-DB (https://covicdb.lji.org/). Negative-stain and cryo-EM structures are available at the Electron Microscopy Data Bank (http://www.emdataresource.org/) under accession codes listed in the key resources table. Additional information required to reanalyze the data reported in this paper is available from the lead contact upon request.

## Acknowledgments

We thank Dr. Ruben Dias Avalos and the LJI Cryoelectron Microscopy Facility for electron microscopy data collection and Dr. Sarah Mudrak (Duke) for program management. We thank Drs. Juan I. Garcia and Colwyn Headley and Mr. Oscar Rodriguez for technical assistance with the *in vivo* studies. We thank the Overton family for launching the study of variants. The authors are indebted to the contributors of samples to the CoVIC study, whose generosity and contributions made a study of this scale possible. We are grateful for INV-0006133 (E.O.S. and B.P.) and INV-008612 and INV-043419 (G.D.T.) from the 10.13039/100000865Bill and Melinda Gates Foundation, 10.13039/100000002NIH U19 AI142790-03S1 (E.O.S.), and the 10.13039/100015595GHR Foundation (E.O.S.) for supporting this study.

## Author contributions

Conceptualization, S.L.S., J.M., B.H., K.M.H., D.B., R.S.B., A.B., L.G., T.G., Y.K., G.D.T., B.P., and E.O.S.; methodology, S.L.S., P.J.H., J.M., B.H., K.M.H., D.B., K.L., J.B.T., S.M.D., R.S.B., A.B., L.G., T.G., Y.K., G.D.T., B.J., and E.O.S.; software, B.H., A.G., D.B., K.L., and CoVIC-DB; validation, S.L.S., P.J.H., J.M., B.H., K.M.H., H.L., D.B., K.L., N.K., J.B.T., J.E.M., M.O.-T., H.M.C., CoVIC-DB, S.M.D., L.M.-S., P.A.P., S.P., B.P., and E.O.S.; formal analysis, S.L.S., P.J.H., J.M., K.M.H., H.L., D.B., K.L., R.H.C.H., G.Q.H., M.A., E.F., N.K., J.B.T., S.M.D., L.M.S., P.A.P., and S.P.; investigation, S.L.S., X.Y., P.J.H., J.M., K.M.H., H.L., D.B., C.T., K.L., N.K., J.B.T., J.E.M., M.O.-T., H.M.C., R.H.C.H., G.Q.H., M.A., E.F., L.M.-S., A.H., C.Y., J.-G.P., B.M., P.A.P., S.P., M.-N.L., N.S.-A., S.K., M. Maddocks, M. Mallory, and T.S.; resources, J.B.T., S.R., A.P., P.K., CoVIC Consortium, R.S.B., A.B., L.G., T.G., Y.K., G.D.T., B.P., and E.O.S.; data curation, S.L.S., J.M., B.H., A.G., CoVIC-DB, B.P., and E.O.S.; writing – original draft, S.L.S., J.M., B.P., and E.O.S.; writing – review & editing, S.L.S., J.M., K.M.H., S.R., P.K., R.S.B., A.B., L.G., G.T., Y.T., G.D.T., B.P., and E.O.S.; visualization, S.L.S., X.Y., J.M., H.L., D.B., and K.L.; supervision, K.M.H., R.B.S., A.B., L.G., T.G., Y.K., G.D.T., B.P., and E.O.S.; project administration, S.L.S., J.B.T., B.P., G.D.T., and E.O.S.; funding acquisition, R.B.D., A.B., L.G., T.G., G.D.T., B.P., and E.O.S.

## Declaration of interests

R.S.B. is a member the VaxArt, Takeda, and Invivyd advisory boards and has collaborative projects with Gilead, J&J, and HilleVax that are unrelated to this work. R.S.B. is a co-inventor of methods and uses of mouse-adapted and derivative SARS-CoV-2 viruses (US patent US11225508B1). D.B. and T.G. are employees of Carterra. Y.K. has received unrelated funding support from Daiichi Sankyo Pharmaceutical; Toyama Chemical; Tauns Laboratories, Inc.; Shionogi & Co. Ltd.; Otsuka Pharmaceutical; KM Biologics; Kyoritsu Seiyaku; Shinya Corporation; and Fuji Rebio.

## STAR★Methods

### Key resources table

**Table:** 

REAGENT or RESOURCE	SOURCE	IDENTIFIER
**Antibodies**		

Human IgG antibodies against SARS-CoV-2 purified using standard methods (CoVIC 1-397)	This study; Hastie et al.^26^, Callaway et al.^25^	https://covic.lji.org
CR3022	Ter Meulen et al.^15^	RRID: AB_3074753
REF-1 (CC12.3) isolated from convalescent SARS-CoV-2 patient	Rogers et al.^42^; Yuan et al.^16^	N/A
REF-1 (CC12.14) isolated from convalescent SARS-CoV-2 patient	Rogers et al.^42^; Yuan et al.^16^	N/A

**Bacterial and virus strains**		

Authentic SARS-CoV-2 virus engineered to express Neon Green protein SARS-CoV-2mNGI	Xie et al.^43^	N/A
Authentic SARS-CoV-2 virus engineered to express a luciferase reporter	Hou et al.^44^	N/A
SARS-CoV-2 D614G/Vesicular Stomatitis Virus pseudovirus	Callaway et al.^25^; Bewley et al.^45^	N/A
SARS-CoV-2 Beta /Vesicular Stomatitis Virus pseudovirus	Callaway et al.^25^; Bewley et al.^45^	N/A
SARS-CoV-2 Delta /Vesicular Stomatitis Virus pseudovirus	Callaway et al.^25^; Bewley et al.^45^	N/A
SARS-CoV-2 BA.1/Vesicular Stomatitis Virus pseudovirus	Callaway et al.^25^	N/A
SARS-CoV-2 BA1.1/Vesicular Stomatitis Virus pseudovirus	Callaway et al.^25^	N/A
SARS-CoV-2 BA.2/Vesicular Stomatitis Virus pseudovirus	Callaway et al.^25^	N/A
SARS-CoV-2/human/USA/WA-CDC-WA-1	BEI Resources	GenBank: MN985325

**Chemicals, peptides, and recombinant proteins**		

SARS-CoV-2 D614G Spike protein full-length ectodomain with HexaPro mutations and C-terminal Foldon, HRV3C protease cleavage site, 8x-His-tag, and strep-tag	Hastie et al.^26^	GenBank: MN908947
SARS-CoV-2 Omicron Beta Spike protein full-length ectodomain with HexaPro mutations and C-terminal Foldon, HRV3C protease cleavage site, 8x-His-tag, and strep-tag	Li et al.^14^	GenBank: QHD43416.1
SARS-CoV-2 Omicron BA.1 Spike protein full-length ectodomain with HexaPro mutations and C-terminal Foldon, HRV3C protease cleavage site, 8x-His-tag, and strep-tag	Callaway et al.^25^	GenBank: QHD43416.1
Soluble RBD	Hastie et al.^26^	GenBank: MN908947
ACE-2	Hastie et al.^26^	UniProt: Q9BYF1
Uranyl formate	Electron Microscopy Sciences	Cat#22451
Papain	Sigma	Cat#P3125
L-cysteine	Calbiochem	Cat#4400

**Critical commercial assays**		

Nano-Glo Luciferase assay	Promega	Cat # N1110
ExpiFectamine CHO Transfection kit	Thermo Fisher	Cat#A29129

**Deposited data**		

Negative stain electron microscopy reconstructions of antibodies in complex with SARS-CoV-2 D614G spike	Electron Microscopy Data Bank (http://www.emdataresource.org/)	24335, 24336, 24337, 24338, 24339, 24340, 24341, 24342, 24343, 24344, 24345, 24346, 24348, 24350, 24351, 24352, 24353, 24354, 24355, 24356, 24357, 24358, 24359, 24360, 24361, 24383, 24384, 24388, 28090, 28091, 28092, 28093, 28094, 28095, 28096, 28097, 28098, 28099, 28100, 28102, 28103, 28104, 28105, 28106, 28168, 28169, 28170, 28171
Data for Affinity, epitope binning, ACE-2 blockage, neutralization data, *in vivo* protection, structural analyses, escape data are deposited in the CoVIC Database, CoVIC-DB	www.covicdb.lji.org	N/A

**Experimental models: Cell lines**		

Vero E6	ATCC	Cat# CRL-1586; RRID:CVCL_0574
Vero E6/C1008	ATCC	Cat# CRL-1586; RRID:CVCL_0059
HEK293T	ATCC	Cat# CRL-3216; RRID:CVCL_0063
ExpiCho-S cells	Thermo Fisher Scientific	Cat# A29127; RRID:CVCL_5J31
Vero	ATTC	Cat# CCL-81 RRID:CVCL_0059
Vero E6	ATCC	Cat# CRL-1586; RRID:CVCL_0574
Vero E6/C1008	ATCC	Cat# CRL-1586; RRID:CVCL_0059
Vero E6/TMPRSS2	BEI Resources	NR-54970

**Experimental models: Organisms/strains**		

K18h-ACE2 mice	Jackson Labs	Cat# 034860

**Oligonucleotides**		

Escape assay CoV-2 Spike 21490 fwd 5'-GGT AGA CTT ATA ATT AGA GAA AAC AAC-3'		This study
Escape assay CoV-2 Spike 25410 rev 5'-TCT CAT AAA CAA ATC CAT AAG TTC GT-3'		This study
Escape assay internal sequencing primer 1 5'-cgt ggt gtt tat tac cct gac-3'		This study
Escape assay internal sequencing primer 2 5'-aca ttc aac tca gga ctt gtt c-3'		This study
Escape assay internal sequencing primer 3 5'-cag ggt ttt tcg gct tta ga-3'		This study
Escape assay internal sequencing primer 4 5'-tgc cct ttt ggt gaa gtt tt-3'		This study
Escape assay internal sequencing primer 5 5'-aga ttg ttt agg aag tct aat ctc aaa-3'		This study
Escape assay internal sequencing primer 6 5'-tga cac tac tga tgc tgt ccg-3'		This study
Escape assay internal sequencing primer 7 5'-tgt agc tag tca atc cat cat tgc-3'		This study
Escape assay internal sequencing primer 8 5'-tca caa at atta cca gat cca tca a-3'		This study
Escape assay internal sequencing primer 9 5'-gaa cca aaa att gat tgc ca-3'		This study
Escape assay internal sequencing primer 10 5'-caa aaa gag ttg att ttt gtg gaa-3'		This study
Escape assay internal sequencing primer 11 5'-aaa tat ttt aag aat cat aca tca cca-3'		This study
Escape assay internal sequencing primer 12 5'-cag act tta ata aca aca tta gta gcg-3'		This study

**Recombinant DNA**		

Empty vector: phCMV3	Genlantis	Cat# P003300
pCAGGS-VSV-G SARS-CoV-2 WT spikeΔCT with luciferase reporter	Kerafast; this study	N/A
pCAGGS-VSV-G SARS-CoV-2 Beta spikeΔCT with luciferase reporter	This study	N/A
pCAGGS-VSV-G SARS-CoV-2 Delta spikeΔCT with luciferase reporter	This study	N/A
pCAGGS-VSV-G	Kerafast	Cat# EH1017
phCMV3-Beta Spike	Callaway et al.^25^	GenBank: QHD43416.1 with L18F, D80A, D215G, D242-244, R246I, K417N, E484K, N501Y, D614G, and A701V mutations
phCMV3-Delta Spike	Callaway et al.^25^	GenBank: QHD43416.1 with T19R, G142D, E156G, D157–158, L452R, T478K, D614G, P681R, and D950N mutations
phCMV3-Omicron BA.1	Callaway et al.^25^	GenBank: QHD43416.1 with A67V, D69/70, T95I, G142D, D143/145, N211I, D212, ins214 EPE, G339D, S371L, S373P, S375F, S477N, T478K, E484A, Q493R, G496S, Q498R, N501Y, Y505H, T547K, D614G, H655Y, N679K, P681H, D796Y, N856K, Q954H, N969K, and L981F mutations
phCMV3-Omicron BA1.1 Spike	Callaway et al.^25^	GenBank: QHD43416.1 with A67V, D69/70, T95I, G142D, D143/145, N211I, D212, ins214 EPE, G339D, R346K, S371L, S373P, S375F, S477N, T478K, E484A, Q493R, G496S, Q498R, N501Y, Y505H, T547K, D614G, H655Y, N679K, P681H, D796Y, N856K, Q954H, N969K, and L981F mutations
phCMV3-Omicron BA.2 Spike	Callaway et al.^25^	GenBank: QHD43416.1 with T19I, L24S, D25/27, G142D, V213G, ins214 EPE, G339D, S371F, S373P, S375F, T376A, D405N, R408S, K417N, N440K, S477N, T478K, E484A, Q493R, Q498R, N501Y, Y505H, D614G, H655Y, N679K, P681H, N764K, D796Y, Q954H, and N969K mutations

**Software and algorithms**		

GraphPad Prism 9	GraphPad Software	https://www.graphpad.com/
Carterra “Kinetics” and “Epitope” software packages	Carterra	https://wwww.carterra-biocom/
NextGen KIT	Carterra	https://wwww.carterra-biocom/
TitrationAnalysis	Li et al.^14^	N/A
Data Analysis HT 12.0 (CFR11) software	Sartorius	http://sartorius.com
CryoSPARC	CryoSPARC	www.cryosparc.com
Chimera X	Pettersen et al.^46^	https://www.cgl.ucsf.edu/chimerax/
ChimeraX-1.8	Pettersen et al.^47^	https://www.cgl.ucsf.edu/chimerax/
SWISS-MODEL	Waterhouse et al.^48^	https://swissmodel.expasy.org/
PHENIX	Adams et al.^49^	https://www.phenix-online.org/
COOT	Emsley et al.^50^	https://www2.mrc-lmb.cam.ac.uk/personal/pemsley/coot/
COCOMAPS	Vangone et al.^51^	https://www.molnac.unisa.it/BioTools/consrank/consrank-nmr.html
PISA	EMBL-EBI^52^	https://www.ebi.ac.uk/pdbe/pisa/
CatBoost	Prokhorenkova et al.^39^ Dorogush et al.^40^	https://catboost.ai/

**Other**		

Carterra LSA	Carterra	https://carterra-bio.com/lsa/
CMDP LSA chip	Carterra	Cat# 4282
HC30M LSA chip	Carterra	Cat# 4279
Octet HTX	Sartorius	http://sartorius.com
Amine reactive 2^nd^ generation (ARG2) biosensor	Sartorius	18-5092
Biacore S200 instrument	Cytiva	https://cytivalifesciences.com
Cytation Hybrid Multi-Mode reader	Biotek Instruments	www.agilent.com
SpectraMax i3x plate reader	Molecular Devices	www.moleculardevices.com
Titan Halo electron microscopy	Thermo Fisher Scientific	www.thermofisher.com
Superose 6 Increase 10/300 GL	GE Healthcare	Cat#29091596
CF400-Cu grids	Electron Microscopy Sciences	Cat# CF400-Cu

### Experimental model and study participant details

#### Study participants

For inclusion in the CoVIC, contributors submitting antibodies isolated from convalescent patients with COVID were required to provide informed consent documentation and evidence that informed consent was obtained. The contributors also provided study protocols and documentation of IRB approval.

#### Mouse strains

Mice were maintained in appropriated animal biosafety level 3 (ABSL3) laboratories at Texas Biomedical Research Institute (Texas Biomed). Female, 6-week-old mice with transgenic expression of human ACE2 receptor under control of the human K18 promoter (K18h-ACE2; The Jackson Labs Cat No. 034860^53^) were used. Mice were observed, clinical signs (hunched back, labored breathing, slow movement, eye discharge, non-responsiveness and/or moribund) were assessed and the animals were weighed daily over a 10-day experimental period. Blood samples (0.1-0.2 mL) were collected after antibody delivery and before virus from the submandibular vein. Sera was collected, frozen at -80°C before shipment to Nexelis for analysis. All experiments involving mice conformed to regulatory standards following the approved Texas Biomed Biosafety (BSC# 20-010) and Institutional Animal Care and Use (IACUC# 1745 MU) Committee approvals.

#### Mammalian cell lines

HEK-293T (ATCC CRL-3216, human, female), Vero (ATCC CCL-81, monkey, female), Vero E6 (ATCC CRL-1586, monkey, female) and Vero E6/TMPRSS2 (BEI Resources NR-54970) cells were cultured in high-glucose Dulbecco’s modified Eagle’s medium containing L-glutamine (DMEM, Invitrogen, Carlsbad, CA) supplemented with 10% fetal bovine serum (Omega Scientific, Tarzana, CA) and 1% penicillin-streptomycin solution. Cells were maintained at 37°C in a humidified atmosphere with 5% CO_2_. ExpiCHO (Chinese hamster, female) cells were cultured in ExpiCHO expression medium (Thermo Fisher) and maintained at 37°C in a humidified atmosphere with 8% CO_2_. Cell lines were not authenticated, but were passaged fewer than 15-20 times after thawing a new vial to prevent phenotypic drift and routinely tested for mycoplasma infection.

### Method details

#### Antibody isolation and purification

Antibodies were isolated and purified using standard methods or as described in Hastie et al.^26^ and Callaway et al.^25^.

#### High-throughput SPR epitope binning using RBD

A classical sandwich assay format was used to determine epitope communities using a Carterra LSA HT-SPR instrument equipped with a CMDP sensor chip. Assays were carried out at 25°C in HBSTE-BSA running buffer (10 mM HEPES pH 7.4, 150 mM NaCl, 3 mM EDTA, 0.05% Tween-20, supplemented with 0.5 mg/ml BSA). Samples were deposited on the sensor chip using two microfluidic modules, a 96-channel print-head (96PH) and a single flow cell (SFC). The chip surface was prepared with 25 mM MES pH 5.5 with 0.05% Tween-20 as a running buffer and activated with a freshly prepared solution of 130 mM 1-ethyl-3-(3-dimethylaminopropyl)carbodiimide (EDC) + 33 mM *N*-hydroxysulfosuccinimide (Sulfo-NHS) in 0.1 M MES pH 5.5 using the SFC. Antibodies (10 μg/mL diluted with 10 mM sodium acetate (pH 4.25)) were immobilized using the 96PH for 10 minutes followed by quenching of unreactive esters with a 7-minute injection of 1 M ethanolamine-HCl (pH 8.5) using the SFC. The array was used for the binning analysis with the HBSTE-BSA buffer as the running buffer and sample diluent. In each cycle, a 4-minute injection of RBD antigen (1.8 μg/mL; 50 nM; aa 318-591 based on GenBank sequence MN908047) was immediately followed immediately by a 4-minute injection of the analyte antibody at 30 μg/mL (200 nM for IgG constructs). After each cycle, the surface was regenerated with double pulses (17 seconds/pulse) of 10 mM Glycine pH 2.0.

Epitope software supplied with the LSA instrument was used to process and analyze the data. Briefly, unprinted locations on the array were used to reference the data and each binding cycle was normalized to the RBD capture level. Analyte antibody binding levels just after the end of the injection was compared to that of a buffer alone injection. Significant increases in signals compared to buffer controls were designated as sandwiches and correspond to non-blocking activity. Heat maps depicting blocking relationships of analyte/ligand pairs were used to visualize competition results. Clones having similar competition patterns cluster together in a dendrogram that was used to assign shared communities. In competition maps, light and dark teal indicate non-blocking and blocking, respectively, and black shading indicates self. Some antibodies could not be regenerated as immobilized ligands, and thus were analyzed only as the analyte. Three reference antibodies were also included in this analysis: CR3022 as well as CC12.3 and CC12.14, (termed REF-1 and REF-2, respectively). CR3022 was isolated from a survivor of SARS and has cross-reactivity with SARS-CoV-2. It targets a cryptic epitope and is a Class 4 antibody.^54^ The neutralizing antibodies CC12.3 (RBD-2a) and CC12.14 (RBD-2c) were isolated from convalescent SARS-CoV-2 patients who were infected early (pre-June 2020) in the pandemic.^16^^,^^42^

#### High-throughput SPR epitope binning using full-length trimeric spike ectodomain

A premix assay format was used to determine epitope communities using a Carterra LSA HT-SPR instrument equipped with a CMDP sensor chip. The data collection and analysis procedure had been reported previously A premix assay format was used to determine epitope communities using a Carterra LSA HT-SPR instrument equipped with a CMDP sensor chip. The data collection and analysis procedure had been reported previously.^14^ Briefly, antibodies were immobilized through amine-coupling. A mixture of antibody and full-length ectodomain D614 HexaPro with a molar ratio of 13.3 (250 nM [37.5 μg/mL] vs. 18.8 nM [10.35 μg/mL]) was incubated for at least 30 minutes and then injected in each cycle, followed by a dissociation step. D614 HexaPro alone was injected periodically for quality testing of activity of immobilized antibodies and for data normalization. Data were first processed with Epitope Tool software (Carterra). After data normalization, the interactions were defined as non-blocking interactions if normalized binding signals just after the end of association were ≤0.7. Two separate binning assays were merged into a single heatmap before carrying out clustering analyses.

#### High-throughput SPR binding kinetics

Binding kinetics measurements for CoVIC antibody constructs were done using the Carterra LSA platform with HC30M sensor chips (Carterra) at 25°C. In each assay, a single analyte was titrated against multiple CoVIC antibody constructs. The CoVIC reference monoclonal antibodies (mAbs) CC12.3 and CC12.14^16^^,^^42^ were included in each assay as positive controls. The binding kinetics data collection and analysis procedures were described previously.^14^^,^^26^ Briefly, antibody constructs were first immobilized onto HC30M chips. Human monoclonal IgG antibodies were captured using anti-Human IgG Fc secondary antibody that was amine-coupled onto the chip; other types of CoVIC antibody constructs (e.g, Fab, scFv, diabodies) were immobilized through amine-coupling directly. Each CoVIC antibody construct was immobilized onto at least 4 separate spots of the same chip, enabling replication of binding kinetics measurements. After immobilization, a two-fold dilution series of the antigen was prepared. The maximum concentrations for RBD and HexaPro constructs were: RBD 40 μg/mL (1.11 μM), D614-HexaPro 100 μg/mL (0.181 μM), D614G-HexaPro 100 μg/mL (0.170 μM), B.1.351-HexaPro 100 μg/mL (0.170 μM),and BA.1-HexaPro 200 μg/mL (0.351 μM). A single antigen was injected in each assay onto the chip surface from the lowest to the highest concentration without regeneration, preceded by blank buffer injections. For each concentration, the data collection time-length for baseline, association and dissociation were 120 seconds, 300 seconds and 900 seconds, respectively. The collected titration data were pre-processed using Kinetics (Carterra) software and then exported and analyzed using the TitrationAnalysis tool. The RBD, NTD and HexaPro construct binding time courses for each antibody construct immobilized on different spots were fitted to a 1:1 Langmuir model to derive k_*a*_, k_*d*_ and K_*D*_ values. For each CoVIC antibody construct-antigen pair, the best triplicate measurements satisfying the preset data acceptance criteria were selected and the averaged k_a_, k_*d*_ and K_*D*_ values are reported. The quality control preset acceptance criteria included: 1) standard error of the estimated k_a_, k_d_ and K_*D*_ in each replicate ≤20%; and 2) fold-change for all 3 parameters within the triplicate ≤ 3.

#### ACE-2 blocking

Biolayer Interferometry (BLI) assays on an Octet HTX instrument (Sartorius) were used to measure the ability of antibodies to block binding of ACE2 to immobilized SARS-CoV-2 RBD. The data collection and analysis procedure for measurement of ACE-2 blocking was described previously.^26^ Briefly, RBD and human serum albumin (HSA) as a reference to subtract response arising from non-specific interactions were immobilized onto amine Reactive 2nd Generation (AR2G) biosensors (Sartorius) through amine coupling. Then sensors loaded with RBD and HSA were in different cycles sequentially dipped into a well plate containing 20 μg/ml antibody solution and then recombinant ACE2 (ACE-2 human IgGFc fusion; 27.5 μg/ml) for 5 minutes each. The binding of ACE2 to immobilized RBD was monitored in the absence and presence of antibodies pre-bound to RBD. Each experiment included mAbs CC12.3 and CC12.14^16^ as reference RBD-binding antibodies and a control SARS-CoV-2 Spike neutralizing mAb (Sino Biological). The data was analyzed using Data Analysis HT 12.0 (CFR11) software (Sartorius). The percent ACE2 blocking was calculated as the percentage of decrease in ACE2 binding for antibodies pre-bound to RBD versus RBD in the absence of antibody. The triplicate averaged signal for ACE2 binding to RBD in the absence of antibody was set as 0% blocking. Triplicate averaged values for ACE-2 blocking percentages are reported if preset data acceptance criterion is satisfied: CV of triplicate measurements was <20% for antibodies having percent ACE2 blocking above 13%, a threshold determined using an influenza hemagglutinin specific mAb.

#### Neutralization assays

##### Pseudovirus with luciferase readout

The fit for purpose pseudotyped virus neutralization assay used by Nexelis^45^ is based on a protocol described by Whitt (2010)^55^ and involves pseudotyped virus particles made using a genetically modified Vesicular Stomatitis Virus from which the glycoprotein G was removed (VSVΔG; Kerafast). The VSVΔG virus is transduced in HEK293T cells previously transfected with the spike glycoprotein of the SARS-CoV-2 coronavirus (Wuhan strain, accession NC_045512) from which the last 19 amino acids of the cytoplasmic tail were removed (ΔCT). The resulting pseudoparticles (VSVΔG – Spike ΔCT) contain a luciferase reporter to provide a signal that can be quantified in relative luminescence units (RLU). Neutralization activity was assessed by 11-point concentration curves (mAb concentrations ranging from 0.004-3.6 μg/mL) from which IC_50_ and IC_90_ values were determined from a four-parameter logistic curve.

##### Pseudovirus with GFP readout

The pseudovirus neutralization assay carried out at La Jolla Institute for Immunology used a previously described protocol.^25^^,^^26^ Briefly, 293T cells were transfected with phCMV3-SARS-CoV-2 S using TransIT according to the manufacturer’s protocol to generate recombinant SARS-CoV-2-pseudotyped VSV-ΔG-GFP virus particles. The cells were washed twice at 24 hr post-transfection with OptiMEM before infection with rVSV-G pseudotyped ΔG-GFP parent virus VSV-G^∗^ΔG-GFP at MOI=2 for 2 hours with rocking. Then, the virus was removed from the cells, which were washed twice with OPTI-MEM containing 2% FBS (OPTI-2) before fresh OPTI-2 was added. The supernatants containing rVSV-SARS-2 were collected at 24 hours post-infection and clarified by centrifugation. To titrate virus, Vero cell monolayers were first generated by seeding cells in 96-well plates at a sufficient density to produce a monolayer at the time of infection. Then, 10-fold serially diluted pseudovirus was added to cells in triplicate wells. The cells were incubated with the pseudovirus at 37°C for 16-18 hr, then fixed with 4% PFA and stained with Hoechst (10 μg/mL) in PBS. After replacing the fixative/stain with PBS, the number of GFP-expressing cells were counted using a CellInsight CX5 imager (ThermoScientific) to quantify the pseudovirus titers, which were expressed as fluorescent forming units, ffu/mL. To measure neutralization, pre-titrated amounts of rVSV-SARS-CoV-2 pseudovirus was incubated with either serially diluted monoclonal antibodies or two standard concentrations (25 μg/mL or 250 ng/mL) for block assays at 37°C for 1 hr and then added to confluent Vero (ATCC CCL-81) monolayers in 96-well plates. The plates were incubated for 16-18 hr at 37°C in 5% CO_2_ before fixation with 4% paraformaldehyde and staining with 10 μg/mL Hoechst. Cells were imaged using a CellInsight CX5 imager and infection was quantified by counting the total number of GFP-expressing cells. Infection was normalized to the average number of cells infected with rVSV-SARS-CoV-2 incubated with human IgG isotype control. Data are presented as the relative infection for each antibody concentration. Neutralization IC_50_ titers were calculated using “One-Site Fit LogIC_50_” regression in GraphPad Prism 9.0. Precision and accuracy of the Saphire lab pseudovirus neutralization assay were evaluated in the SARS-CoV-2 neutralization assay concordance survey (SNACS), ranking among the highest for specificity, precision and accuracy.

##### Authentic virus with mNeonGreen readout

Neutralization of authentic SARS-CoV-2 by all mAbs in the CoVIC panel was assessed using a fully infectious virus engineered to express Neon Green protein (SARS-CoV-2-mNGl).^43^ The replication properties of this virus are similar to that of the original virus yet allow high-throughput assessment of neutralization with a readout that is more reliable than that achieved with traditional plaque reduction neutralization tests (PRNTs) that involve manual counting of plaques and are less easily adapted to a 96-well plate form. Vero E6 cells were pre-seeded in 96-well black plates with clear bottoms the day before infection. CoVIC monoclonal antibodies ranging from 200-0.0002 μg/mL (11-point concentration curve) were pre-incubated with SARS-CoV-2-mNG (3 or fewer passages) at MOI 0.005 PFU per cell for 1 hr in U-bottom 96-well plates in the BSL-3 containment. Then, the media was replaced with 100 μl virus-antibody mixtures and incubated for 48 hr. The levels of neutralization was evaluated based on the intensity of mNeon-Green fluorescence, which reflects virus infection, using a high-throughput imaging reader at 488 nm as previously described.^56^ Neutralization curves were generated from which IC_50_ and IC_90_ values were determined. Neutralization activities measured by PRNT and with high-throughput SARS-CoV-2mNG microneutralization assay were shown to be comparable (R2=0.90) to the results obtained with the mNG reporter.

##### Authentic virus with luciferase readout

Neutralization of authentic SARS-CoV-2 carrying D614G, B.1.351, B.1.1.7 and other coronaviruses by mAbs in the CoVIC panel was assessed using a method like that previously described with minor modifications.^44^ Under BSL-3 containment, serially-diluted mAbs at 8 concentrations are incubated with 800 PFU/well nLuc virus for one hour at 5% CO2 and 37°C. After incubation, the virus/antibody mixtures are added in duplicate to black-walled 96-well plates containing Vero E6/C1008 cells (2 x 10^4^ cells/well). Each plate also contains virus-only control wells. The plates are incubated for 24 hr at 37°C, 5% CO_2_ and the cells are lysed before measurement of luciferase activity with the Nano-Glo Luciferase Assay System (Promega) according to the manufacturer’s instructions. Neutralization activity is expressed as the concentration at which the observed relative light units (RLU) are reduced by 50% relative to virus-only control wells.

#### Epitope mapping via negative stain EM

Negative stain electron microscopy (nsEM) was used to determine the structure of complexes between full length spike ectomains and CoVIC antibodies existing in various antibody formats, including IgG, Fab, scFv and VHH. Either IdeS (Promega) or papain (Sigma) were used to generate antibody Fab fragments, which were purified by ion exchange chromatography using a MonoQ column (GE).

To form complexes, 140 μg purified HexaPro.D614G spike (or the indicated Variant of Concern) in TBS was incubated overnight at room temperature with Fab (70 μg), VHH (50 μg), scFv (70 μg) or IgG (140 μg). Then, size exclusion chromatography (SEC) with a Superdex 6 Increase column (GE) was used to purify the spike-antibody complexes, which were verified by SDS-PAGE. The purified complexes (4μL; ∼0.02 mg/mL) were applied to a CF400-Cu negative-stain grid (Electron Microscopy Sciences) that was then stained with 0.75% uranyl formate (Electron Microscopy Sciences). A Titan Halo electron microscopy (Thermo Fisher) equipped with a Falcon 3EC direct electron detector was used to collect between 50 and 400 micrographs for each sample at a magnification of 58,000X. CryoSPARC^57^ was used to reconstruct EM-Maps, which were aligned and displayed using Chimera X.^58^

#### Spike-Fab complex structure determination by cryo-EM

Antibody complexes were obtained by incubating spike protein with ∼3 molar excess of Fabs at room temperature. 3 μL of the sample for cryogenic electron microscopy (cryo-EM) imaging were prepared by applying the complex solution to Quantifoil-2/1 grids (Electron Microscopy Sciences), followed by blotting and plunge-freezing into liquid ethane using a Vitrobot Mark IV (Thermo Fisher Scientific).

TEM images were collected automatically using EPU on a Titan Krios 300 kV electron microscope (Thermo Fisher Scientific) at a magnification of 75,900 with a Gatan K3 detector for a total dose of ∼50 e-/Å^2^. Data processing was performed using Cryosparc v3.3.1.^57^ Movies were motion-corrected by Patch motion correction. CTF estimation was performed using Patch CTF estimation. Particles were first picked using the CryoSPARC blob picker, then those particles selected after 2D classification were used to train Topaz,^59^ a neural network for further particle picking. Picked particles were extracted and subjected to rounds of 2D classification for selection.

For the CoVIC-154 Fab and SARS-CoV-2 spike complex, the reconstruction was obtained by homogenous refinement using an Ab-initio model as a reference, followed by local CTF refinements and non-uniform refinement in CryoSPARC. Reported resolutions are based on the gold-standard Fourier shell correlation (FSC) of 0.143 criteria. The models of SARS-CoV-2 spikes (PDB: 6VXX), and homology models of antibody Fab generated using SWISS-MODEL^48^ were docked into the corresponding reconstructions using Chimera.^46^ The models were refined using PHENIX real space refine^49^ and COOT.^50^ The final models were validated using the MolProbity server.^60^ Structural analysis was performed using COCOMAPS,^51^ and PISA.^52^ Figures were generated using ChimeraX-1.8.^47^

#### *In vivo* model of SARS-CoV-2 infection

The protective efficacy of a subset of CoVIC antibodies was tested in female, 6-week-old K18h-ACE2 mice (n=10/antibody; avg wt. 20g) with transgenic expression of human ACE2 receptor under control of the human K18 promoter.^53^ Upon recording the body weight, mice were first given the indicated antibody intraperitoneally at either 0.5 or 1.5 mg/kg. The 0.5 mg/kg dose was used early in the study and was then updated to 1.5 mg/kg. The dose was selected based on a dose titration study with the reference antibody CC12.3.^16^ The selected dose of 1.5 mg/kg was based on the dose at which 40-60% of animals survived at the end of the 10-day period. The negative control group (n=5) and the control group for infection (n=5) were intraperitoneally inoculated with 1X PDB (non-treated-non-infected group, non-treated-infected group, respectively), and the control treatment group was intraperitoneally injected with the reference antibody CC12.3 (REF-1) at 1.5 mg/kg. At 24 hours after antibody delivery (or after delivery of PBS in the case of the control groups), blood samples (0.1=0.2 mL) were collected from the mice via the submandibular vein. The mice were then challenged intranasally (25 μL/nostril) with 1.0 x 10^5^ PFU SARS-CoV-2/human/USA/WA-CDC-WA-1 (GenBank MN985325) at passage 6. Next generation sequencing was used to confirm that the virus stocks were 100% identical to the original BEI Resources P4 stock. Working stocks were also confirmed to lack the Bristol deletion and other deletions/mutations. The non-treated, non-infected group was intranasally inoculated with 1X PBS (25 μL/nostril). Serum was collected from blood samples, preserved at -80°C, and shipped to Nexelis for analyses. Mice were observed, clinical signs were assessed and the animals were weighed daily over a 10-day experimental period. Any animal that lost ≥25% body weight and/or showed any clinical sign was humanely euthanized.

#### Escape mutation analysis

To test the risk that viruses will “escape” antibody neutralization, virus (500 plaque-forming units (PFU) of an early Wuhan SARS-CoV-2 isolate) was incubated with 2-fold dilutions of antibodies (dilution series of 10 μg/mL to 0.02 μg/mL (lower if needed)) at 37°C for 1 hour. Media was removed from Vero E6/TMPRSS2 cells cultured in 96-well plates and the cells were incubated with the virus-antibody mixtures. Viral amplification was assessed by cell rounding and death relative to uninfected cells that were not treated with antibody. When cell death levels reach 80% or 7 days -post-infection, virus (p1) was harvested from the wells with the highest concentration of mAb used to neutralize. Samples from all wells were also harvested and retained for analysis if needed.

Then, the harvested virus (p1) was passaged again and incubated with antibody at the same concentration at which the virus was harvested p1 as well as four higher antibody concentrations. Cells were again monitored for virus infection/cell death and virus (p2) was harvested at 7 days-post-infection of if cell death reached 80%. Virus was harvested from the highest antibody concentration used for neutralization, titrated and ∼100 PFU was incubated at 37°C for 1 hour with four different concentrations starting with the antibody concentration at which p2 virus was harvested or virus with no antibody. After incubation, the cells were washed to remove unbound virus, and 1.0% MCM was added. Plaques were allowed to form and 10 individual plaque-purified escaped viruses were isolated from the highest antibody concentration for which plaque formation occurred. Viruses were amplified in Vero E6/TMPRSS2 cells to generate a stock of potential escape mutant viruses.

To confirm that the viruses could no longer be neutralized by the specific mAb, ∼100 PFU of virus was incubated with antibody at the appropriate concentration or without antibody. Cells were infected and grown for three days in the absence or presence of the appropriate antibody. Cell death was monitored and cell culture supernatants were harvested to determine virus titers. If a virus was not neutralized by antibody, virus titers were similar between virus incubated with and without antibody. The associated spike gene from these viruses was amplified using primers flanking the spike gene open reading frame, and sequenced using 12 internal sequencing primers to identify mutations.

If no resistant viruses arise after p2, virus continued to be passaged as described above in the presence of antibody for up to five passages. Since the SARS-CoV-2 replication complex does have an associated proofreading mechanism, no mutations in spike occurred in some instances.

### Quantification and statistical analysis

#### Calculation of Spearman’s correlation coefficient

All values of the Spearman’s rank correlation coefficient mentioned in this study were calculated using the ‘spearmanr’ function within the Python package SciPy.^61^

#### Regression analysis

The performance of individual and combinations of antibody features in predicting *in vivo* protection was calculated by training the Categorical Boosting algorithm (CatBoost)^39^^,^^40^ using five-fold cross-validation. In this scheme, the data was shuffled and split into 5 folds, in which one-fold was used as a test dataset while the remaining four were used for training. The trained regression model was then used to predict the *in vivo* protection of the held-out test dataset and the Spearman’s correlation calculated between the predicted and observed values. This process was repeated five times, with each of the remaining folds being assigned as the test set in each step. The five different values of the correlation were averaged to obtain the mean value. A max-min normalization was conducted separately on the training and test data for each of the five steps prior to training. The hyperparameters for the CatBoost regression model were: number of iterations=50, depth of decision tree=3, learning rate=0.1, loss function = root mean square error (RMSE).

### Additional resources

Data described in this study are publicly available at the CoVIC database at https://covicdb.lji.org/. This study did not involve any clinical trials.

## Supplemental information

Document S1. Figures S1–S5

Table S1. CoVIC epitope community, *in vivo* survival, neutralization, affinity, and ACE-2 blockage data, related to Figures 1, 2, 3, and 4

Table S2. Antibody species, isotype, and sequence information, related to Figures 1 and 3

Document S2. Article plus supplemental information
