# Supplementary material for: A global collaboration for systematic analysis of broad-ranging antibodies against the SARS-CoV-2 spike protein
Source: Cell Rep. 2025 Apr 2;44(4):115499. doi: 10.1016/j.celrep.2025.115499 (PMC12014896; doi:10.1016/j.celrep.2025.115499)
Supplement: Document S1. Figures S1–S5 [file mmc1.pdf]

Cell Reports, Volume *44*

Supplemental information

A global collaboration for systematic analysis of broad-ranging antibodies against the SARS-CoV-2 spike protein

Sharon L. Schendel, Xiaoying Yu, Peter J. Halfmann, Jarjapu Mahita, Brendan Ha, Kathryn M. Hastie, Haoyang Li, Daniel Bedinger, Camille Troup, Kan Li, Natalia Kuzmina, Jordi B. Torrelles, Jennifer E. Munt, Melissa Maddocks, Mary Osei-Twum, Heather M. Callaway, The CoVIC-DB Team, Stephen Reece, Anne Palser, Paul Kellam, S. Moses Dennison, Richard H.C. Huntwork, Gillian Q. Horn, Milite Abraha, Elizabeth Feeney, Luis Martinez-Sobrido, Paula A. Pino, Amberlee Hicks, Chengjin Ye, Jun-Gyu Park, Billie Maingot, Sivakumar Periasamy, Michael Mallory, Trevor Scobey, Marie-Noelle Lepage, Natalie St-Amant, Sarwat Khan, Anaïs Gambiez, Coronavirus Immunotherapeutic Consortium, Ralph S. Baric, Alexander Bukreyev, Luc Gagnon, Timothy Germann, Yoshihiro Kawaoka, Georgia D. Tomaras, Bjoern Peters, and Erica Ollmann Saphire

SUPPLEMENTAL FIGURES AND TABLES

Supplemental Figure 1 (Related to Figures 1-6). **Workflow of the CoVIC.** Contributing groups first downselected antibodies for submission based on antibody features including affinity for spike, ACE-2 blocking activity, and/or neutralization activity. All antibodies at minimum demonstrated nanomolar affinity for spike protein (either full-length or RBD). The antibodies were then shipped to La Jolla Institute for Immunology (LJI), where they were assigned a code name known only to the contributor and the CoVIC program manager. The antibodies were aliquoted under the code names and identical sets were shipped to nine different partner labs that carried out the indicated assay. The partner labs deposited the data into the publicly available CoVIC database (CoVIC-DB), which is housed at LJI. Nexelis and LJI carried out assays of pseudovirus neutralization using luciferase (PNV-L) and GFP (PNV-L) reporters, respectively. UTMB and UNC carried out neutralization of authentic virus using mNeonGreen (Auth-M) and luciferase (Auth-L) reporters, respectively. UTMB: University of Texas Medical Branch at Galveston; UNC: University of North Carolina at Chapel Hill; Texas Biomed: Texas Biomedical Research Institute.

Supplemental Figure 2 (Related to Figure 2). **Percentage of ACE-2 blockage by CoVIC antibodies. (A)** Antibodies are arranged according to RBD and FL communities. Numbers represent the percentage blockage of ACE-2 binding to immobilized SARS-CoV-2 RBD induced by the indicated antibody. **(B)** Box plot of the percentage blockage of ACE-2 binding to immobilized by CoVIC antibodies arranged by FL community. Mean values are at the intersection of the darker and lighter-shaded regions, which represent the lower and upper quartile, respectively. Whiskers extend to 1.5-times the interquartile range. Circles correspond to individual CoVIC antibodies and are colored according to the RBD community.

Supplemental Figure 3 (Related to Figure 4). Affinity of antibodies that retained pan-spike binding affinity. **(A)** Box plot of binding affinities for antibodies that had binding affinity for D614G, Beta and Omicron. Mean values are at the intersection of the darker and lighter-shaded regions, which represent the lower and upper quartile, respectively. Whiskers extend to 1.5-times the interquartile range. Circles correspond to individual CoVIC antibodies and are colored according to the FL community. Median dissociation constant (K_D (M)) values for **(B)** RBD and **(C)** full-length spike ectodomain among RBD (upper) and full length (FL; lower) epitope communities. Affinity for full-length spike ectodomain was determined for Wuhan-Hu1 (W) as well as Beta (B) and Omicron (O) variants of concern is also shown, as is the fold-change in affinity with respect to Wuhan-Hu1. The percentage value corresponds to the number of antibodies that exhibited affinity for the indicated antigen. The epitope groups having the highest and lowest affinity are highlighted in blue and salmon, respectively, and the minimum, maximum and median values for all the epitope groups are shown in gray at the bottom of the table.

Supplemental Figure 4 (Related to Figure 5). Representative negative-stain electron microscopy (NS-EM) structures show a variety of epitope footprints and binding mechanisms. NS-EM structures of CoVIC panel antibodies in complex with full-length spike ectodomain were determined and the epitope footprint is shaded according to the epitope community determined with soluble RBD. The full-length epitope bin is listed in the upper right-hand corner. Side (left) and top (right) views of the spike protein are shown. Structures were determined using full-length IgG, except for those with an asterisk by the CoVIC ID for which Fab or ScFv were used. Black dots indicate antibodies that had bivalent binding that was associated with retention of neutralization activity against Omicron and its subvariants BA1.1 and BA.2.

Supplemental Figure 5 (related to Figure 6). Results of escape mutation assay highlighted by full-length epitope. (A) Table is as shown in Figure 6 with the top row listing all amino acid positions at which mutations were detected. “X” indicates that the residue was mutated in a variant, while filled squares indicate that the detected mutation was within 1 or 2 residues of an amino acid that was mutated in a variant. The rows at the

bottom of the table show mutations reported in public databases (e.g., GSAID). **(B)** Bubble plot is as in Figure 6, except circle colors correspond to those defined by epitope binning using full-length spike ectodomain. The circle size corresponds to the number of antibodies that were affected by the mutation. The residue numbers radiate outward from the lowest residue number in the center. **(C)** Detected escape mutations.

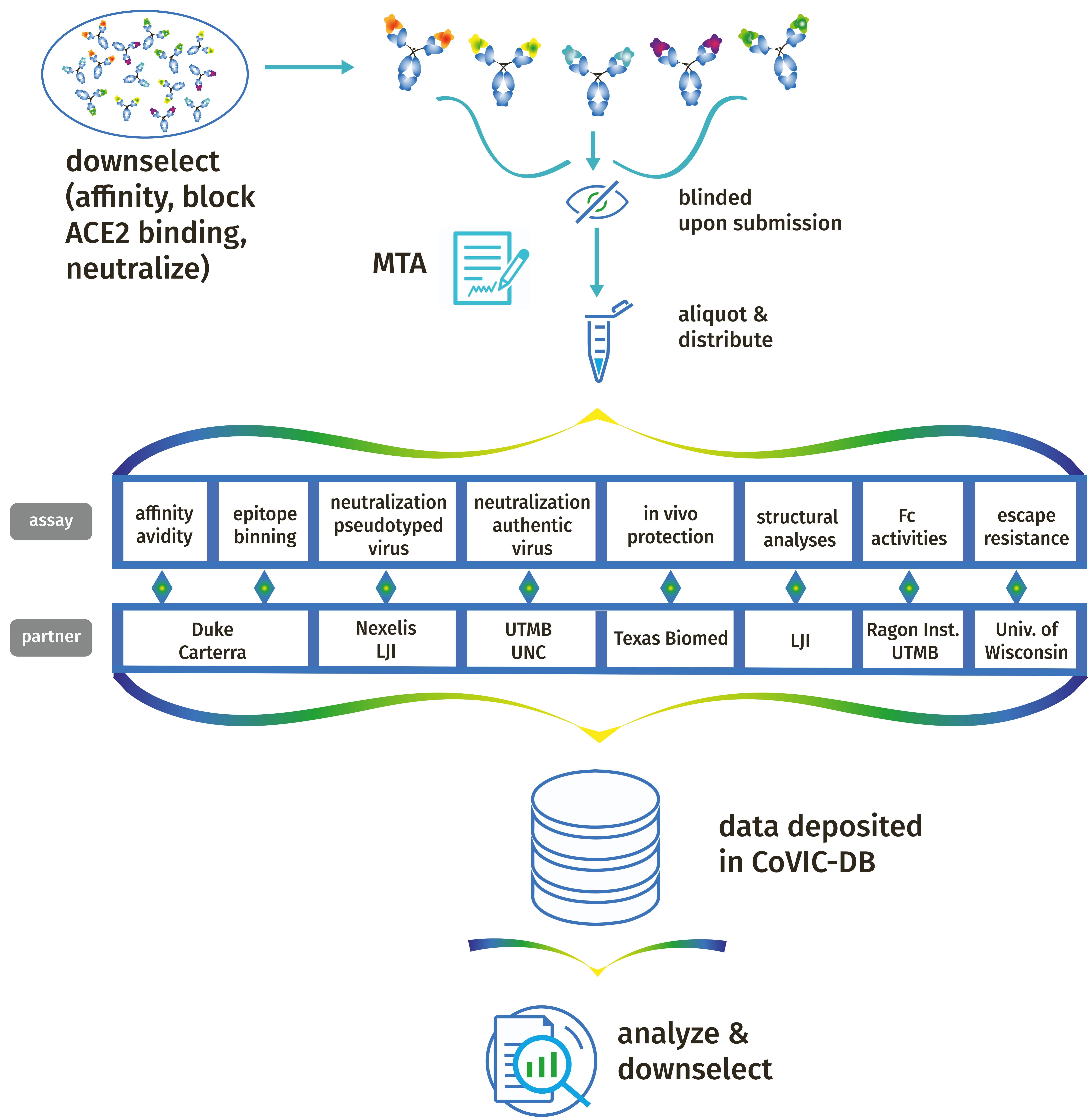
downselect (affinity, block ACE2 binding, neutralize)
MTA
blinded upon submission
aliquot & distribute
assay
affinity avidity
epitope binning
neutralization pseudotyped virus
neutralization authentic virus
in vivo protection
structural analyses
Fc activities
escape resistance
partner
Duke Carterra
Nexelis LJI
UTMB UNC
Texas Biomed
LJI
Ragon Inst. UTMB
Univ. of Wisconsin
data deposited in CoVIC-DB
analyze & downselect

Supplemental Figure 2

A

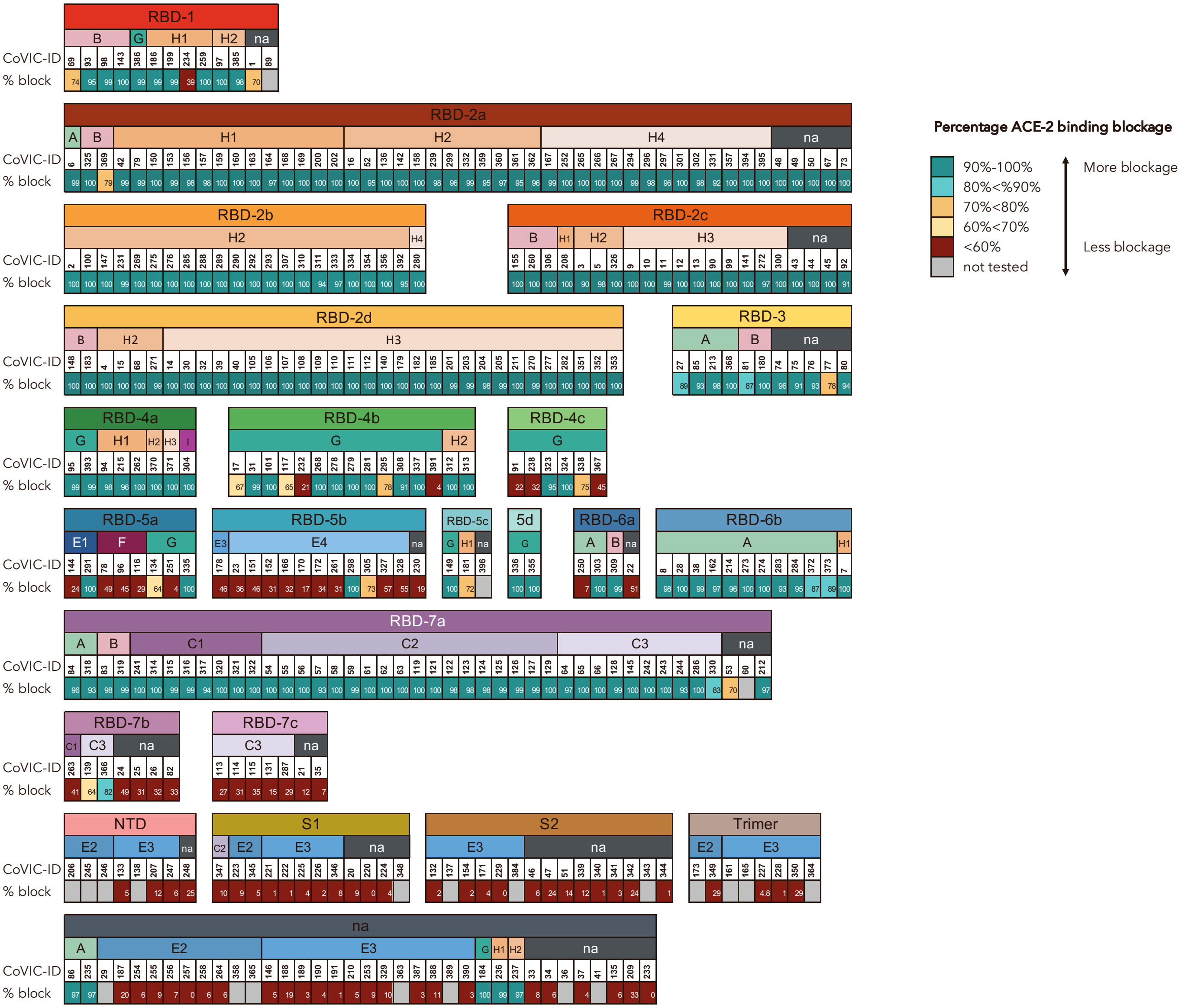

B

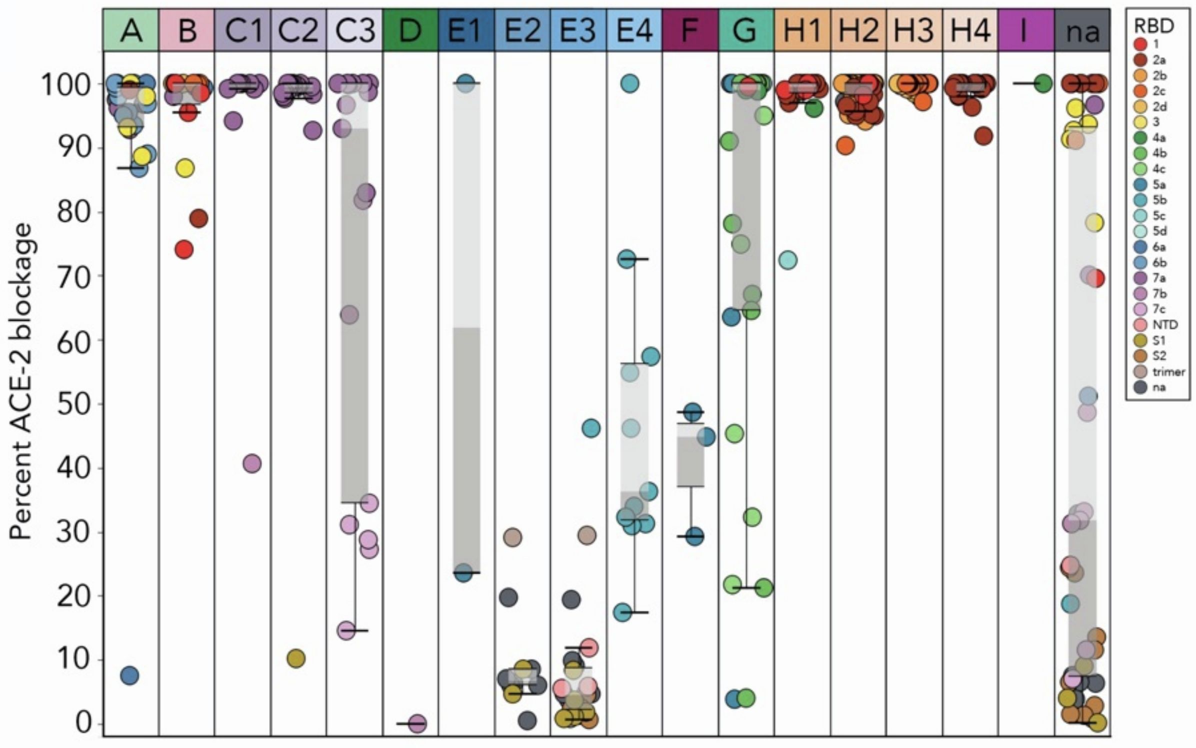

Supplemental Figure 3

A

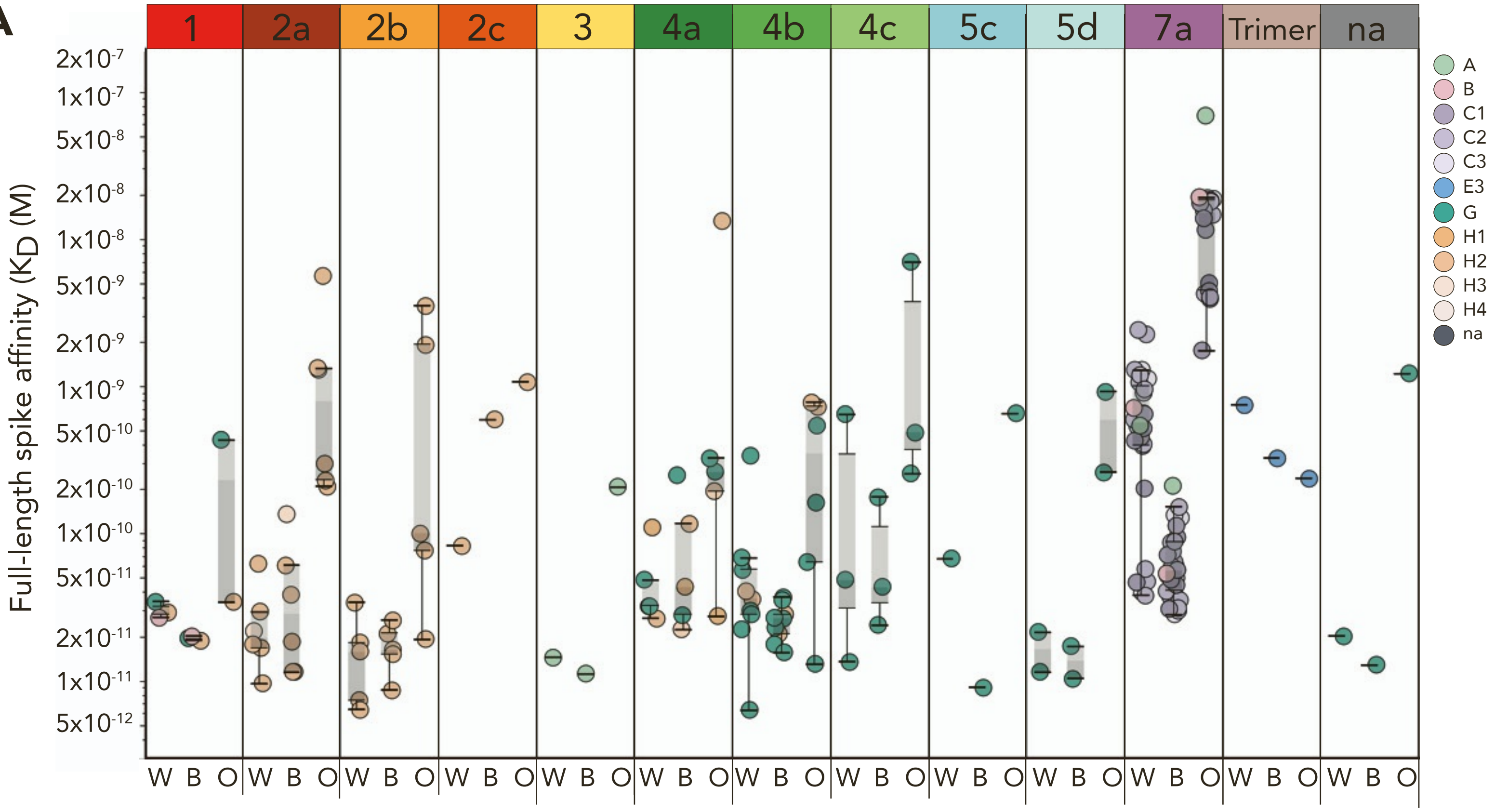

B

Total	RBD Comm	RBD Median KD (M)	n	%
13	1	5.40E-09	12	92%
49	2a	4.70E-09	48	98%
22	2b	2.90E-09	22	100%
21	2c	5.60E-09	21	100%
34	2d	2.10E-09	34	100%
11	3	1.30E-07	11	100%
8	4a	2.90E-09	8	100%
15	4b	1.50E-09	15	100%
6	4c	1.70E-09	6	100%
8	5a	9.60E-09	8	100%
13	5b	6.30E-09	13	100%
3	5c	1.20E-08	2	67%
2	5d	7.30E-09	2	100%
4	6a	9.10E-09	4	100%
12	6b	7.30E-10	12	100%
43	7a	3.00E-09	41	95%
7	7b	2.70E-08	7	88%
7	7c	8.50E-10	7	100%
8	NTD	na	0	0%
12	S1	na	0	0%
15	S2	na	0	0%
8	trimer	na	0	0%
36	na	3.00E-08	6	17%
	Min	7.3E-10		
	Max	1.3E-07		
	Median	5.4E-09		

Total	FL Comm	RBD Median KD (M)	n	%
22	A	3.9E-09	22	100%
16	B	5.0E-09	16	100%
9	C1	3.0E-08	9	100%
19	C2	1.8E-07	18	95%
17	C3	2.6E-07	17	100%
2	E1	3.5E-09	2	100%
17	E2	na	0	0%
35	E3	7.5E-08	2	6%
11	E4	6.3E-09	11	100%
3	F	9.2E-08	3	100%
29	G	2.3E-09	29	100%
25	H1	6.4E-09	24	96%
46	H2	2.3E-09	46	100%
39	H3	1.8E-09	39	100%
16	H4	1.9E-09	16	100%
1	I	2.0E-07	1	100%
50	na	7.9E-08	25	50%
	Min	1.8E-09		
	Max	2.6E-07		
	Median	6.4E-09		

C

Total	RBD Comm	Full-length W Median KD (M)	n	%	Full-length B Median KD (M)	Fold-change B vs. W	n	%	Full-length O Median KD (M)	ΔW/O	Fold-change O vs. W	n	%
13	1	9.6E-11	12	92%	4.2E-11	2.3	9	69%	8.9E-10	0.1071	9	5	38%
49	2a	9.8E-11	49	100%	4.1E-10	0.2	49	100%	2.7E-08	0.0036	280	30	61%
22	2b	2.1E-11	22	100%	1.6E-11	1.3	22	100%	3.7E-09	0.0058	173	17	77%
21	2c	4.4E-11	21	100%	5.3E-10	0.1	14	67%	1.4E-08	0.0032	310	7	33%
34	2d	1.2E-11	34	100%	6.2E-10	0.0	23	68%	4.1E-08	0.0003	3475	18	53%
11	3	8.4E-10	11	100%	2.6E-10	3.2	11	100%	5.6E-08	0.0151	66	9	82%
8	4a	4.1E-11	8	100%	8.0E-11	0.5	8	100%	3.0E-10	0.1372	7	6	75%
15	4b	4.1E-11	15	100%	2.8E-11	1.4	15	100%	7.3E-10	0.0562	18	12	80%
6	4c	9.1E-11	6	100%	1.0E-10	0.9	6	100%	8.9E-10	0.1017	10	6	100%
8	5a	8.3E-11	8	100%	4.2E-11	2.0	8	100%	2.8E-09	0.0299	33	6	75%
13	5b	5.9E-10	13	100%	2.4E-10	2.4	13	100%	4.5E-09	0.1299	8	13	100%
3	5c	5.0E-11	2	67%	1.8E-11	2.8	2	67%	6.6E-10	0.0765	13	1	33%
2	5d	1.7E-11	2	100%	1.4E-11	1.2	2	100%	2.0E-11	0.825	1	2	100%
4	6a	1.5E-09	4	100%	2.4E-10	6.3	4	100%	2.0E-08	0.0773	13	4	100%
12	6b	1.1E-10	12	100%	8.3E-11	1.3	12	100%	5.6E-10	0.1956	5	3	25%
43	7a	5.0E-10	39	91%	5.7E-11	8.7	42	98%	1.5E-08	0.0327	31	26	60%
7	7b	1.9E-09	7	100%	7.5E-10	2.5	7	100%	2.7E-08	0.0699	14	5	71%
7	7c	3.8E-10	7	100%	2.2E-10	1.7	7	100%	5.9E-09	0.064	16	6	86%
8	NTD	5.7E-10	7	88%	8.4E-10	0.7	5	63%	2.4E-08	0.0235	42	2	25%
12	S1	1.8E-10	11	92%	3.4E-10	0.5	8	67%	1.9E-09	0.0987	10	6	50%
15	S2	4.3E-10	8	53%	1.9E-10	2.3	6	40%	1.1E-09	0.4024	2	9	60%
8	Trimer	8.8E-10	8	100%	3.0E-10	2.9	4	50%	1.1E-08	0.0803	12	5	63%
36	na	4.4E-11	33	92%	3.5E-10	0.1	21	58%	6.5E-08	0.0007	1493	14	39%
	Min	1.2E-11			1.4E-11				2.0E-11				
	Max	1.9E-09			8.4E-10				5.6E-08				
	Median	1.0E-10			2.0E-10				4.1E-09				

Total	FL Comm	Full-length W Median KD (M)	n	%	Full-length B Median KD (M)	Fold-change B vs. W	n	%	Full-length O Median KD (M)	ΔW/O	Fold-change O vs. W	n	%
22	A	2.6E-10	22	100%	8.3E-11	3.08	22	100%	2.3E-08	1.1E-02	90.6	13	59%
16	B	1.4E-10	16	100%	9.4E-11	1.44	14	88%	9.4E-09	1.4E-02	69.2	11	79%
9	C1	2.0E-10	7	78%	5.7E-11	3.56	9	100%	9.0E-09	2.3E-02	44.2	8	89%
19	C2	6.0E-10	19	100%	5.4E-11	11.17	19	100%	1.5E-08	4.1E-02	24.5	7	37%
17	C3	2.5E-10	17	100%	1.3E-10	2.00	17	100%	8.8E-09	2.8E-02	35.3	13	76%
2	E1	6.6E-11	2	100%	7.3E-11	0.91	2	100%	3.2E-10	2.1E-01	4.9	2	100%
17	E2	4.7E-11	17	100%	4.2E-10	0.11	10	59%	4.7E-08	1.0E-03	997.9	1	10%
35	E3	7.9E-10	33	94%	4.9E-10	1.61	23	66%	3.9E-09	2.0E-01	4.9	21	91%
11	E4	6.8E-10	11	100%	2.3E-10	2.94	11	100%	4.5E-09	1.5E-01	6.7	11	100%
3	F	5.7E-11	3	100%	4.7E-11	1.20	3	100%	2.6E-08	2.2E-03	450.5	1	33%
29	G	4.9E-11	29	100%	3.1E-11	1.56	29	100%	8.5E-10	5.8E-02	17.4	25	86%
25	H1	1.3E-10	25	100%	3.8E-10	0.33	20	80%	3.6E-08	3.5E-03	284.1	8	40%
46	H2	3.2E-11	46	100%	2.4E-11	1.35	44	96%	3.2E-09	9.9E-03	100.9	34	77%
39	H3	1.1E-11	39	100%	4.6E-10	0.02	27	69%	4.0E-08	2.8E-04	3598.2	31	115%
16	H4	4.9E-11	16	100%	3.8E-10	0.13	16	100%	4.0E-08	1.2E-03	809.8	13	81%
1	I	1.2E-10	1	100%	2.3E-10	0.53	1	100%	1.0E-09	1.2E-01	8.4	1	100%
50	na	1.1E-09	36	72%	8.6E-10	1.30	28	56%	2.6E-08	4.4E-02	22.9	21	75%
	Min	1.1E-11			2.4E-11				3.2E-10				
	Max	1.1E-09			8.6E-10				4.7E-08				
	Median	1.3E-10			1.3E-10				9.4E-09				

W: Wuhan-Hu1
B: Beta (B.1.351)
O: Omicron (BA.1)

Supplemental Figure 4

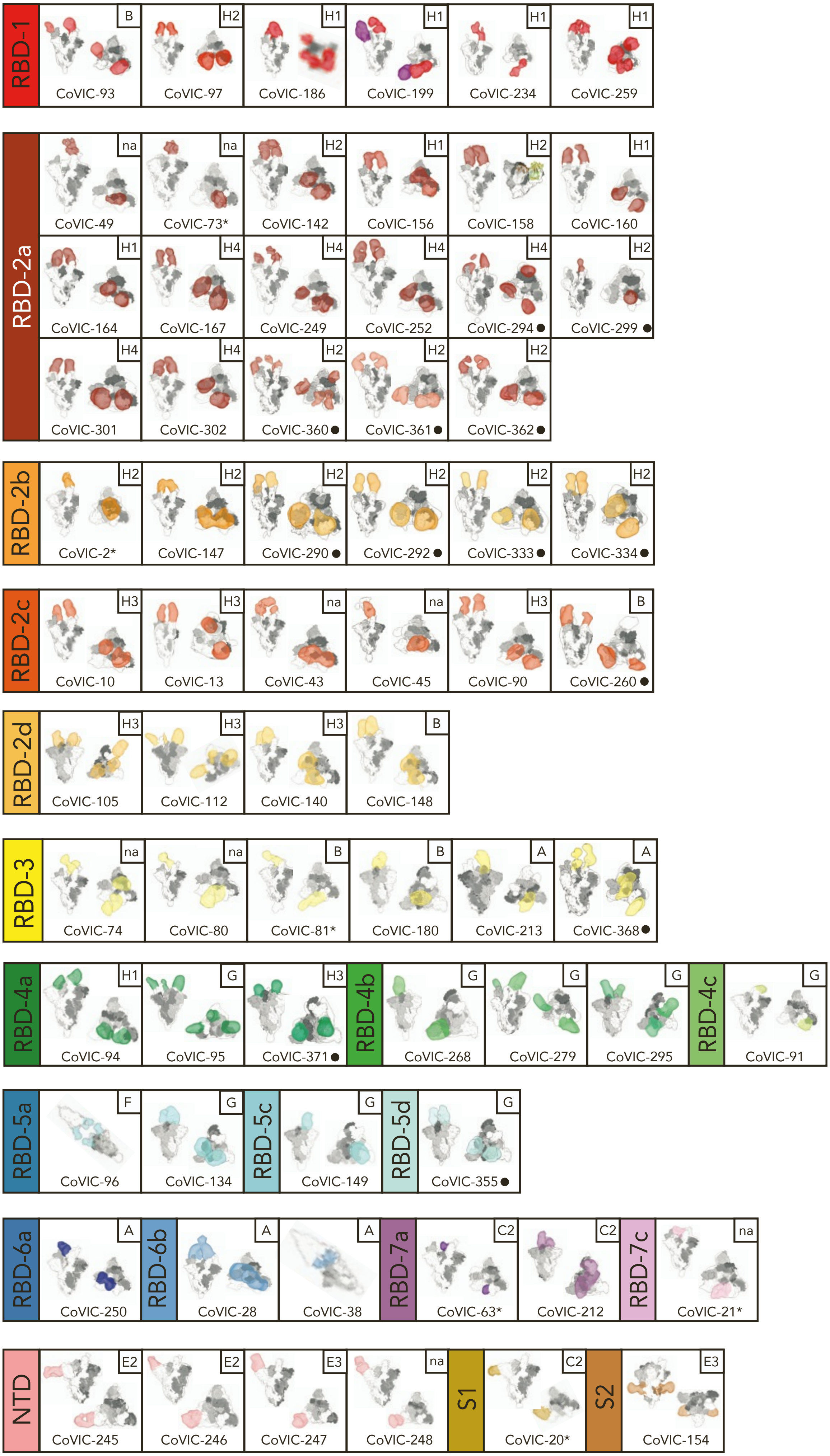

Supplemental Figure 5

A

ESCAPE MUTATIONS DETECTED

VoC	Pango Lineage	67	Δ68-9	Δ68-70	70	Δ140	Δ141	Δ144	Δ141-4	Δ241-7	339	348	369	371	Δ374	375	384	408	417	420	440	442
G614	B.1	A			V						G	A	Y	S		S	P	R	K	D	N	D
Alpha	B.1.17		X	X				X														
Beta	B.1.351									■									X			
Gamma	P.1					■													X			
Delta	B.1.617.2					■	■	■	■													
Omicron	BA.1	X	■	■	■	■	■	■	■		X		■	X	■	X			X		X	X
Omicron	BA.1.1	X	■	■	■	■	■	■	■		X	■	■	X	■	X			X		X	■
Omicron	BA.2					■	■	■	■		X		■	X	■	X		X	X		X	■
Omicron	BA.2.12.1					■	■	■	■		X		■	X	■	X		X	X		X	■
Omicron	BA.5	■	■	■	■	■	■	■	■		X		■	X	■	X		X	X		X	■
Omicron	BQ.1.1	■	■	■	■	■	■	■	■		X	■	■	X	■	X		X	X		X	■
Omicron	XBB.1.5					■	■	X	■		X	■	■	X	■	X		X	X		X	■
Omicron	XBB.1.16					■	■	X	■		X	■	■	X	■	X		X	X		X	■
Omicron	EG.5.1					■	■	X	■		X	■	■	X	■	X		X	X		X	■
Omicron	BA.2.86	■	■	■	■	■	■	X	■		X		■	X	■	X		X	X		X	■
Omicron	JN.1	■	■	■	■	■	■	X	■		X		■	X	■	X		X	X		X	■
Omicron	XEC	■	■	■	■	■	■	X	■		X		■	X	■	X		X	X		X	■
Omicron	KP.3.1.1	■	■	■	■	■	■	X	■		X		■	X	■	X		X	X		X	■
Omicron	LP.1	■	■	■	■	■	■	X	■		X		■	X	■	X		X	X		X	■
Any mutation occurred		V, S			I, M, F						D, H, R	A, T, S		F, L		F		S, N	N, T, R		K, R	

VoC	Pango Lineage	444	447	448	449	Δ449	450	Δ450	459	460	470	475	481	484	Δ484	485	486	487	489	490
G614	B.1	K	G	N	N		N		S	N	T	A	N	E		G	F	N	Y	F
Alpha	B.1.17																			
Beta	B.1.351													X	■					
Gamma	P.1													X	■					
Delta	B.1.617.2						■	■												
Omicron	BA.1	X	X									■		X	■	■	■			
Omicron	BA.1.1	■	■									■		X	■	■	■			
Omicron	BA.2											■		X	■	■	■			
Omicron	BA.2.12.1						■	■				■		X	■					
Omicron	BA.5						■	■				■		X	■	■	X	■		
Omicron	BQ.1.1	X					■	■	■	X		■		X	■	■	X	■		
Omicron	XBB.1.5	■	■	■					■	X		■		X	■	■	X	■	■	X
Omicron	XBB.1.16	■	■						■	X		■		X	■	■	X	■	■	X
Omicron	EG.5.1	■	■						■	X		■		X	■	■			■	X
Omicron	BA.2.86	■	■	■	■	■	X		■	X		■	X	X	■	■	X	■		
Omicron	JN.1	■	■	■	■	■	X	■	■	X		■	X	X	■	■	X	■		
Omicron	XEC	■	■	■	■	■	X	■	■	X		■	X	X	■	■	X	■		
Omicron	KP.3.1.1	■	■	■	■	■	X	■	■	X		■	X	X	■	■	X	■		
Omicron	LP.1	■	■	■	■	■	X	■	■	X		■	X	X	■	■	X	■		
Any mutation occurred		R, N, T					D, K, I			I, K, H	I, N		Y, K	K, A, Q, R, V, S			V, S, P, A, I, L		H	S, P

VoC	Pango Lineage	491	493	494	496	501	504	508	571	588	591	654	675	682	685	703	839	1023	1029	1118	1181	1195
G614	B.1	P	Q	S	G	N	G	Y	D	K	S	E	Q	R	R	N	D	N	M	D	K	E
Alpha	B.1.17					X			■					■						X		
Beta	B.1.351					X										■						
Gamma	P.1					X						■							■			
Delta	B.1.617.2													■								
Omicron	BA.1	■	X	■	X	X	■					■		■								
Omicron	BA.1.1	■	X	■	X	X	■					■		■								
Omicron	BA.2	■	X	■	■	X	■					■		■								
Omicron	BA.2.12.1	■	X	■	■	X	■					■		■		■						
Omicron	BA.5				■	X	■					■		■								
Omicron	BQ.1.1				■	X	■					■		■								
Omicron	XBB.1.5	■	X	■	■	X	■					■		■								
Omicron	XBB.1.16	■	X	■	■	X	■					■		■								
Omicron	EG.5.1	■	X	■	■	X	■					■		■								
Omicron	BA.2.86				■	X	■		■			■		■								
Omicron	JN.1				■	X	■		■			■		■								
Omicron	XEC	■	X	■	■	X	■		■			■		■								
Omicron	KP.3.1.1	■	X	■	■	X	■		■			■		■								
Omicron	LP.1	■	X	■	■	X	■		■			■		■								
Any mutation occurred			R, K	P, T	S	X, Y, T			A	N		D, V, K, A, Q	H, R, K	A, W		I, S	Y	Y		V, H, Y	I	Q

X Mutation occurred in variant of concern (VoC)
■ Mutation is adjacent (within 1-2 residues) of detected escape mutation

B

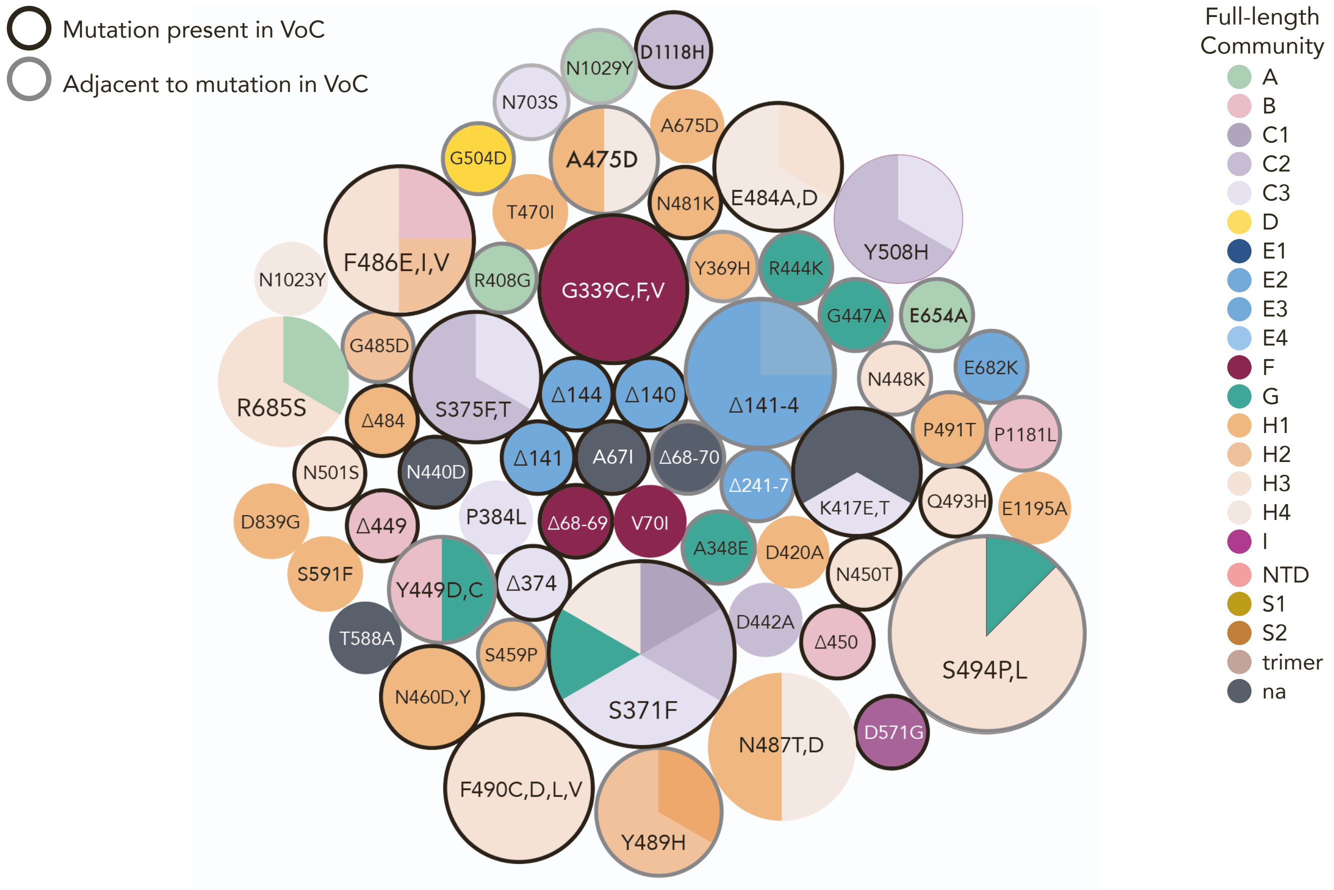

C

CoVIC ID	Epitope (FL)	Escape mutation	CoVIC ID	Epitope (FL)	Escape mutation	CoVIC ID	Epitope (FL)	Escape mutation	CoVIC ID	Epitope (FL)	Escape mutation
COVIC-2	H2	Y489H	COVIC-78	F	Δ68-69; V70I	COVIC-168	H1	N460D	COVIC-248	na	A67I
COVIC-30	H3	E484A, D; F486V; N501S	COVIC-84	A	R408G; E654A	COVIC-169	H1	D839G	COVIC-249	H4	N1023Y
COVIC-32	H3	N448K, N450T; S494L, P; G496V	COVIC-93	B	R685S	COVIC-179	H3	T470I	COVIC-250	A	N1029Y
COVIC-36	na	Δ68-70	COVIC-96	F	G339C	COVIC-180	B	P1181L	COVIC-253	E3	Δ141
COVIC-40	H3	F490C, V	COVIC-101	G	S494L	COVIC-189	E3	Δ140; Δ141-144	COVIC-255	E2	Δ144; E682K
COVIC-45	na	K417T	COVIC-116	F	G339C, F, V	COVIC-191	E3	Δ141-144	COVIC-256	E2	Δ241-247
COVIC-47	na	T588A	COVIC-147	H2	G485D	COVIC-201	H3	F486I; F490L	COVIC-259	H1	E1195A
COVIC-50	na	K417E	COVIC-149	G	A348E	COVIC-220	na	N440D	COVIC-260	B	Δ449; Δ450; Y449D
COVIC-52	H2	Y489H	COVIC-153	H1	S459P	COVIC-221	E3	G504D	COVIC-265	H4	A475D; E484A; N487D
COVIC-54	C2	S375F; D1118H	COVIC-155	B	F486E	COVIC-241	C1	S371F	COVIC-267	H4	S371F
COVIC-59	C2	S371F	COVIC-156	H1	N481K; Δ484; N487T	COVIC-242	C3	S371F; P384L	COVIC-268	G	R444K; G447A; Y449C
COVIC-61	C2	D442A	COVIC-157	H1	D420A; A475D; A675D	COVIC-243	C3	N703S	COVIC-269	H2	F486I
COVIC-63	C2	Y508H	COVIC-159	H1	P491T; S591F	COVIC-244	C3	S371F; S375T; Y508H			
COVIC-64	C3	Δ374; Y508H; D571G	COVIC-160	H1	Y369H; N460Y; Y489H; N487T	COVIC-246	E2	Δ141-144			
COVIC-65	C3	K417E	COVIC-167	H4	N487T	COVIC-247	E3	Δ141-144			
